# Long-Term Exposure to Traffic-Related Air Pollution and Diabetes: A Systematic Review and Meta-Analysis

**DOI:** 10.3389/ijph.2023.1605718

**Published:** 2023-05-31

**Authors:** Meltem Kutlar Joss, Hanna Boogaard, Evangelia Samoli, Allison P. Patton, Richard Atkinson, Jeff Brook, Howard Chang, Pascale Haddad, Gerard Hoek, Ron Kappeler, Sharon Sagiv, Audrey Smargiassi, Adam Szpiro, Danielle Vienneau, Jennifer Weuve, Fred Lurmann, Francesco Forastiere, Barbara H. Hoffmann

**Affiliations:** ^1^ Swiss Tropical and Public Health Institute, Allschwil, Switzerland; ^2^ University of Basel, Basel, Switzerland; ^3^ Institute for Occupational, Social and Environmental Medicine, Centre for Health and Society, Medical Faculty, University of Düsseldorf, Düsseldorf, Germany; ^4^ Health Effects Institute, Boston, MA, United States; ^5^ Department of Hygiene, Epidemiology and Medical Statistics, School of Medicine, National and Kapodistrian University of Athens, Athens, Greece; ^6^ Population Health Research Institute, St. George’s University of London, London, United Kingdom; ^7^ Occupational and Environmental Health Division, Dalla Lana School of Public Health, University of Toronto, Toronto, ON, Canada; ^8^ Department of Biostatistics and Bioinformatics, Rollins School of Public Health, Emory University, Atlanta, Georgia; ^9^ Institute for Risk Assessment Sciences, Utrecht University, Utrecht, Netherlands; ^10^ Center for Environmental Research and Children’s Health, Division of Epidemiology, School of Public Health, University of California, Berkeley, Berkeley, CA, United States; ^11^ Department of Environmental and Occupational Health, School of Public Health, University of Montreal, Montreal, QC, Canada; ^12^ Department of Biostatistics, University of Washington, Seattle, WA, United States; ^13^ Department of Epidemiology, Boston University School of Public Health, Boston, MA, United States; ^14^ Sonoma Technology, Inc., Petaluma, CA, United States; ^15^ Faculty of Medicine, School of Public Health, Imperial College, London, United Kingdom

**Keywords:** diabetes, particulate matter, traffic-related air pollution, NO_2_, confidence assessment

## Abstract

**Objectives:** We report results of a systematic review on the health effects of long-term traffic-related air pollution (TRAP) and diabetes in the adult population.

**Methods:** An expert Panel appointed by the Health Effects Institute conducted this systematic review. We searched the PubMed and LUDOK databases for epidemiological studies from 1980 to July 2019. TRAP was defined based on a comprehensive protocol. Random-effects meta-analyses were performed. Confidence assessments were based on a modified Office for Health Assessment and Translation (OHAT) approach, complemented with a broader narrative synthesis. We extended our interpretation to include evidence published up to May 2022.

**Results:** We considered 21 studies on diabetes. All meta-analytic estimates indicated higher diabetes risks with higher exposure. Exposure to NO_2_ was associated with higher diabetes prevalence (RR 1.09; 95% CI: 1.02; 1.17 per 10 μg/m^3^), but less pronounced for diabetes incidence (RR 1.04; 95% CI: 0.96; 1.13 per 10 μg/m^3^). The overall confidence in the evidence was rated moderate, strengthened by the addition of 5 recently published studies.

**Conclusion:** There was moderate evidence for an association of long-term TRAP exposure with diabetes.

## Introduction

Diabetes is a major metabolic disease characterized by persistent hyperglycemia if untreated [1]. According to the International Diabetes Federation (IDF), 537 million adults are living with diabetes worldwide with an estimated 45% who are undiagnosed. By 2045, 783 million adults are projected to have diabetes. The most common form of diabetes, type 2, accounts for approximately 90% of cases. Type 2 diabetes is characterized by insulin resistance, a diminished response to insulin of cells in the muscles, liver and fat [2]. Apart from genetic factors that contribute to diabetes risk, the most familiar risk factors include behaviors such as lack of physical activity and diet. Environmental exposures, such as air pollution are also expected to play a role [3].

In 2019, 19.9% of diabetes-related deaths and 19.6% of the diabetes-related disability-adjusted life-years (DALY) were attributed to particulate air pollution [4]. Several systematic reviews have concluded that ambient air pollution is associated with diabetes mellitus [5, 6], diabetes type 1 [7] or gestational diabetes mellitus [8]. Understanding how diabetes risk is affected by air pollution from specific sources informs useful air quality policies and other interventions. Automotive vehicular traffic is a prevalent source of air pollution, especially in cities. In animal studies, traffic-related air pollution (TRAP) was shown to elicit oxidative stress and subclinical inflammation, resulting in impaired insulin signaling and insulin resistance [9]. The sole systematic review to date evaluating the association of TRAP exposure with diabetes concluded there was a positive association between the two [10]. TRAP is a complex mixture and includes tailpipe and non-tailpipe emissions. Tailpipe emissions, from combustion of fossil fuels, contain particulate matter (PM), particularly as elemental carbon (EC) or soot, and nitrogen oxides. Non-tailpipe emissions originate from brake, tire, and road surface abrasion, and re-suspension of dust [11] and include PM trace metals such as copper (Cu), iron (Fe) and zinc (Zn). In high-income countries, non-tailpipe emissions comprise over half of the PM from traffic [12].

The Health Effects Institute (HEI) appointed an expert Panel to systematically evaluate the epidemiological evidence on the associations between TRAP and selected health outcomes including mortality, respiratory diseases, birth outcomes, and cardiometabolic health effects including diabetes. The resulting HEI Special Report was published in 2022 [13], along with a short communication paper of the main findings [14].

Here, we elaborate in depth on the findings and confidence assessment on TRAP in relation to effects on diabetes in adults, and in supplemental analyses we extend our interpretation to include evidence published after completion of the original literature search.

## Methods

The 2022 review was led by an expert Panel of 13 experts in environmental sciences, epidemiology, exposure assessment and statistics, supported by an external team and HEI staff. We used a systematic approach to search and select the literature for inclusion in the review, assess study quality, summarize results, and assess the confidence in the association between TRAP and diabetes. The methods were based on standards set by Cochrane Collaboration [15], the World Health Organization [16], and the National Institute of Environmental Health Sciences Office of Health Assessment and Translation (NIEHS OHAT) [17] and are described in more detail in the special report [13]. The protocol was published [18] and registered in PROSPERO 2019 CRD42019150642 available from: https://www.crd.york.ac.uk/prospero/display_record.php?ID=CRD42019150642.

### Exposure Framework for TRAP

Pollutants emitted by motorized traffic are also emitted by other (combustion) sources. A novel framework to formalize the process of determining whether the air pollution exposure contrast in a study was dominated by traffic, we developed a novel framework [18]. In brief, the framework combined three aspects of TRAP assessment and results from a study had to entail all three aspects to be included: 1) Included studies used measures of defined traffic-related pollutants and/or indirect traffic measures, such as distance to major roads or traffic density. Eligible pollutants were NO_2_, NO_x_, NO, carbon monoxide (CO), EC (including related metrics such as black carbon, black smoke, and PM absorbance), ultrafine particles (UFP), non-tailpipe PM trace metals [e.g., copper (Cu), iron (Fe) and Zinc (Zn)], polycyclic aromatic hydrocarbons (PAHs), benzene, PM_10_, PM_2.5_ and PM_coarse_ (Supplementary Table S1). 2) Both the pollution surface and participants’ addresses in the included studies had to meet the framework’s thresholds for spatial resolution (e.g., 5 km grid). 3) Eligible exposure assessment methods included appropriate models or surface monitoring at sufficient spatial resolutions (Supplementary Table S2).

Following this framework, we excluded studies on short-term (minutes to months) effects or self-reported exposures to TRAP. We included studies that assigned individual-level exposure based on models exploiting within-city (i.e., neighborhood) contrasts, that were considered to stem primarily from traffic. Studies that exclusively used between-city contrasts were excluded. In general, the larger the study area, the less likely a measured or modelled contrast in pollution stems primarily from traffic emissions. Therefore, epidemiological studies in larger regions (e.g., state- or country-wide studies) were only included when they adjusted for area in their analysis. PM is generally not specific to traffic. We included results pertaining to PM measures (aerodynamic diameter ≤10 µm [PM_10_] or ≤2.5 µm [PM_2.5_]) in certain settings, e.g., urban areas, so long as they met more stringent requirements for inclusion. For example, PM studies based exclusively on surface monitoring were excluded, but studies using chemical transport models, dispersion models or land-use regression models with a resolution finer or equal to 5 km were included.

To specify how well the studies met the multiple criteria of the exposure framework, we defined an indicator for high traffic specificity based on even stricter criteria. We used this indicator for sensitivity analyses. High traffic specificity was mainly assigned to models with finer resolution (<1 km) or PM models considering only traffic-specific sources/emissions also with a resolution <1 km.

We converted effect estimates for pollutants expressed as ppb or ppm to μg/m³, or mg/m³ using standard WHO scaling factors (standardization of units). For example, 1 ppb NO_2_ = 1.88 μg/m³, assuming an ambient pressure of 1 atm and a temperature of 25°C [19]. Effect estimates for black carbon (BC), black smoke (BS) and PM_2.5_ absorption (soot) were converted into EC-equivalent estimates [20, 21].

### Search Strategy

We performed a systematic literature search in PubMed and the specialized LUDOK (Literature database and services on Health Effects of Ambient Air Pollution https://www.swisstph.ch/en/projects/ludok/datenbanksuche/) database matching the PECOS (Population, Exposure, Comparator, Outcome and Study) question [15] for epidemiologic studies:

“In the adult population (P), what is the increase in risk of prevalence and incidence of diabetes (O) per unit increase (C) of long-term exposure to traffic-related air pollution (E), observed in studies relevant for the health outcome and exposure duration of interest (S).”

We searched the databases from 1 January 1980 through 31 July 2019. This end date was chosen *a priori* for the comprehensive HEI special report comprising dozens of exposures and health outcomes. The search strategy was based on a review protocol developed by the NIEHS OHAT (OHAT) and further refined using a combination of medical subheadings (MeSH) and keywords (Supplementary Table S3). The search strategy was supplemented with hand-searches of references in recent reviews. These were identified by the original search, an additional search in the LUDOK database or individual bibliographic databases curated by HEI and Panel members.

### Eligibility Criteria

We applied the following inclusion and exclusion criteria according to the predefined PECOS statement. Studies needed to be published in English in a peer-reviewed journal.

#### Population

We included studies reporting on the general human adult population, aged 18 and older, from all geographical areas were included. We excluded studies reporting on occupational exposure or exclusively indoor settings as they would be difficult to compare with general population outdoor exposures.

#### Exposure

Studies that assessed long-term exposure (months to years) to TRAP as defined in the exposure framework were included.

#### Comparator

Studies analyzing health effects of TRAP either on a continuous scale or in exposure categories and reporting a quantitative measure of association plus a measure of precision were included.

#### Outcome

Eligible studies evaluated the incidence or prevalence of diabetes, and defined diabetes as fasting blood glucose levels above a threshold, self-reported physician-diagnosed diabetes, clinical diagnosis (ICD-9: 250, ICD-10: E10–E14) in medical records or claims, or the use of blood glucose-lowering medication.

#### Study Design

We included original epidemiologic studies with individual level data adopting a cohort, case–cohort, case–control, cross-sectional, or intervention design.

We excluded studies that: analyzed only area-level data, evaluated effects of short-term exposure (e.g., time-series or case cross-over studies), reported only unadjusted results, showed clear evidence of an analytical error, were strictly methodological of focused on gene-environment interactions.

### Study Selection

We used DistillerSR, a web–based, systematic review software program version 2.29.8 [22], for screening, data extraction and risk of bias assessment. Initial screening based on title and abstract was done by two independent reviewers. Secondary screenings of study eligibility, especially regarding the exposure criterion, were conducted by two independent reviewers based on the full-text, supplements and related exposure assessment papers. At this full-text review stage, the reviewers documented reasons for excluding any given study (Supplementary Table S4). Any disagreement on inclusion was resolved by discussion.

### Risk of Bias

We assessed risk of bias (RoB) in the estimation of all exposure–outcome associations that were included in the meta-analyses. We used a modified version of the tool developed for the risk of bias assessment in systematic reviews for the WHO Air Quality Guidelines [16, 23]. In brief, the risk of bias tool guides the assessment of each study’s potential for bias from six domains and related subdomains of systematic error sources: 1) confounding; 2) selection bias; 3) exposure assessment; 4) outcome measurement; 5) missing data; and 6) selective reporting. Most domains have subdomains. The risk of bias for each subdomain and for each domain overall was given a rating of low, moderate or high. No summary classification was derived across the domains.

### Meta-Analysis

We conducted meta-analysis for each exposure-outcome pair where three or more studies reported results; we separately analysed findings from incidence and prevalence studies. Effect estimates from single-pollutant models were selected for the meta-analysis. For presenting results on each pollutant, we applied a uniform pollutant contrast to all contributing estimates and the resulting meta-analytic summary estimate (e.g., RR per 10 μg/m^3^ increment in NO_2_), which necessitated converting some contributing estimates (see Supplementary Eq. S1). We chose the contrast of a given pollutant to reflect a realistic range of exposures in most studies, by using the pollutant concentration increments from a large European ESCAPE study [24]. Meta-analysis was not conducted for the exposure metrics related to distance and density of traffic, because the varying definitions across the studies precluded such analyses. We computed summary effect estimates with random effects models, using restricted maximum likelihood to estimate the between study variance [25]. Random effects models were chosen *a priori* because of the expected differences in effect estimates related to differences in populations and pollution mixtures. Statistical heterogeneity was assessed using primarily I^2^, where I^2^ values of <50% were interpreted as low; between 50% and 75% as moderate; and >75% as high degree of heterogeneity [26]. The risk estimates hazard ratio (HR), relative risk (RR), incidence rate ratio (IRR) and odds ratio (OR) were considered to approximate the risk ratio [27] and were therefore analysed together as done previously [28]. We use the general term RR to indicate any of the ratio measures.

If a sufficient number of studies were available, we performed additional meta-analyses to assess consistency of the association by: geographic regions; level of risk of bias (selection bias, missing data, confounding, exposure assessment, outcome assessment); smoking adjustment; traffic specificity; and adjustment for the co-exposure noise. All analyses and plots were done with the statistical program R (version 3.6.0), using the libraries “metafor” (v.2.4‐0), “meta,” (v. 4.16‐2), “forestplot” (v.1.10.1), “ggplot” (v. 3.3.3).

### Assessment of the Evidence

We assessed: 1) the quality of the body of evidence using a modified OHAT protocol [17], which itself is based on the GRADE (Grading of Recommendations Assessment, Development and Evaluation) approach; and 2) the confidence in an association between TRAP and diabetes in a “narrative” assessment. These complementary methods are described fully in the HEI Special Report, Additional Materials 5.3 [13]. We also reflect on the confidence assessment in a separate paper (under review).

For studies included in meta-analyses, we conducted the quality assessments separately for each pollutant and study design. Starting with a confidence rating depending on study design (moderate for cohort studies and low for cross-sectional studies), the rating was then downgraded for factors that decrease confidence (high RoB, unexplained inconsistency, imprecision, and publication bias) and upgraded for factors that increase confidence in the body of evidence (monotonic exposure-response, consistency across populations, and consideration of residual confounding). We did not consider the downgrading factor “indirectness” because we included only studies of human exposure to TRAP in direct association with diabetes. Furthermore, we did not use the upgrading factor “large magnitude of effect,” because this factor was unlikely to be meaningful. This *a priori* decision was based on experiences in the WHO systematic reviews of air pollution, where large or very large effect sizes (i.e., large RR > 2 or very large RR > 5 as defined in OHAT) never occurred [30, 31]. Large RRs were also not observed in our review (Supplementary Figure S1). Next, evaluations per pollutant were combined across study designs, and then across pollutants which was informed by the pollutant with the highest rating.

Since the OHAT assessment is geared toward studies entering a meta-analysis and focusses on the quality of the body of evidence rather than the presence of an association, the Panel also conducted a more inclusive “narrative” assessment. This additionally considered, e.g., pollutants with less than three studies reporting results or those studying indirect traffic measures. While many of the same aspects relevant to evidence synthesis were included in both assessments, there were some subtle differences, most notably regarding the magnitude and direction of the association, and the consistency across pollutants and indirect traffic measures.

In both assessments we rated the level of confidence as high, moderate, low or very low. The two approaches were considered complementary and combined into an overall confidence assessment.

### Updated Search and Supplemental Analyses

To interpret results of our original review (indicated in tables and figures as “Global 2022”) in light of evidence published after the ending date of this review’s literature search, we repeated the search for eligible studies, starting from June 2019 up to May 2022. Studies identified in this new search were not incorporated into the risk of bias and confidence assessment. However, we incorporated their findings into supplemental meta-analyses to investigate the robustness of our original meta-analytic results to the inclusion of recently published evidence (indicated in tables and figures as “Global 2023”).

## Results

### Study Selection

The search strategy for all health outcomes considered for the comprehensive review yielded 13,660 unique articles. After initial screening, exclusion of studies not meeting the inclusion criteria, and restricting to articles on diabetes outcomes, we identified 45 studies, 21 of which entered this review after full-text assessment (Table 1, Supplementary Figure S2: PRISMA flow chart). Most studies were excluded, because the spatial scale of the pollution surface or participants’ address did not meet the criteria (Supplementary Table S4).

**TABLE 1:** Characteristics of the studies reporting on the association of traffic-related air pollution and diabetes incidence or prevalence (Global 2022).

References	Study name	Location	Study period	Study design in analysis	Sample size N (% women)	Age at baseline	Ascertainment of diabetes	Confounder adjusted for	Results (estimate^a^, 95% CI, increment)
[45]	DDCH	Copenhagen and Aarhus, Denmark	1993–2006	Cohort	51,818 (53%)	56	Disease register	Age, sex, iSES, smoking, behavior^b^, BMI	Incidence
									NO_2_ 1.04 (1.00, 1.08) per 4.9 μg/m^3^ ^c^
									NO_x_ 1.02 (1.00, 1.04) per 11.4 μg/m^3^ ^c^
									Distance 1.07 (0.95, 1.21) <50 vs. >50 m
									Density 1.02 (1.00, 1.04) per 1,200 vehicle-km/day
[40]	ONPHEC	Toronto, Canada	1996–2012	Cohort	1,056,012 (53%)	51	Administrative data from hospital and insurance registries	Age, sex, nSES, comorbidities^d^	IncidenceNO_2_ 1.06 (1.05, 1.07) per 4.0 ppb^c^PNC 1.06 (1.05, 1.08) per 9948.4 particles/cm^3^
[41]	British Columbia Diabetes Cohort	Vancouver, British Columbia, Canada	1994–2002	Cohort	380,738 (54%)	58	Administrative data from insurance registry	Age, sex, nSES	Incidence
									NO_2_ 1.00 (0.98, 1.02) per 8.4 μg/m^3^ ^c^
									NO 1.04 (1.01, 1.05) per 13.13 μg/m^3^
									PM_2.5abs_ 1.03 (1.01, 1.04) per 0.9 1e-5/m^c^
									PM_2.5_ 1.03 (1.01, 1.05) per 1.6 μg/m^3^ ^c^
[35]	BWHS	Los Angeles, California, United States	1995–2005	Cohort	39,922 (100%)	39	Doctor-diagnosed	Age, iSES, nSES, smoking, behavior, BMI, familial diabetes	IncidenceNO_x_ 1.25 (1.07, 1.46) per 12.4 ppb^c^
[36]	BWHS	United States	1995–2013	Cohort	430,032 (100%)	39	Doctor-diagnosed	Age, iSES, nSES, smoking, behavior, BMI, area, questionnaire cycle	IncidenceNO_2_ 0.90 (0.82, 1.00) per 9.7 ppb^c^
[63]	Hoorn Diabetes Screening	West Friesland, Netherlands	1998–2000	Cross sectional	8018 (51%)	Range: 50–75	Multimodal^e^	Age, sex, nSES, (BMI)^f^	Prevalence
									NO_2_ 1.03 (0.82, 1.31) 14.2–15.2 vs. 8.8–14.2 μg/m^3^
									NO_2_ 1.25 (0.99, 1.56) 15.2–16.5 vs. 8.8–14.2 μg/m^3^
									NO_2_ 0.80 (0.63, 1.02) 16.5–26 vs. 8.8–14.2 μg/m^3^
									Distance 0.88 (0.70, 1.13) 2–74 vs. 220–1,610 m
									Distance: 1.17 (0.93, 1.48) 74–140 vs. 220–1,610 m
									Distance: 1.12 (0.88, 1.42) 140–220 vs. 220–1,610 m
									Density: 1.09 (0.85, 1.38) 882–2007 vs. 63–516 thousand vehicles/day
									Density: 1.13 (0.89, 1.44) 680–882 vs. 63–516 thousand vehicles/day
									Density: 1.25 (0.99, 1.59) 516–680 vs. 63–516 thousand vehicles/day
[44]	Plovdiv Diabetes Survey	Plovdiv, Bulgaria	2014–2014	Cross sectional	513 (61%)	36	Doctor-diagnosed	Age, sex, iSES, smoking, behavior, BMI, familial diabetes, noise	PrevalencePM_2.5_ 1.32 (0.28, 6.24) >25 vs. <25 μg/m^3^PAH (BaP) 1.76 (0.52, 5.98) >6 vs. <6 ng/m^3^
[34]	SAPALDIA	Multiple cities, Switzerland	2002–2002	Cross sectional	6,392 (52%)	52	Multimodal	Age, sex, iSES, nSES, smoking, behavior, BMI, area	PrevalenceNO_2_ 1.21 (1.05, 1.39) per 10 μg/m^3^ ^c^PM_10_ 1.44 (1.21, 1.71) per 10 μg/m^3^ ^c^
[33]	SAPALDIA	Multiple cities, Switzerland	2002–2011	Cohort	2,631 (52%)	53	multimodal	Age, sex, iSES, nSES, smoking, behavior, BMI, area	IncidenceNO_2_ 0.92 (0.67, 1.26) per 15 μg/m^3^ ^c^
[42]	CANHEART	Ontario, Canada	2008–2008	Cross sectional	2,496,458 (52%)	53	Disease register	Age, sex, iSES, nSES, area	PrevalenceNO_2_ 1.16 (1.14, 1.17) per 10 ppb^c^
[37]	SALIA	North Rhine-Westphalia, Germany	1985–2006	Cohort	17,752 (100%)	54	Multimodal	Age, sex, smoking, BMI	IncidenceNO_2_ 1.42 (1.16, 1.73) per 15 μg/m^3^ ^c^PM_2.5abs_ 1.27 (1.09, 1.48) per 0.39 1e-5/m^c^Distance 2.54 (1.31, 4.91) (low education) < 100 vs. >100 mDistance 0.92 (0.58, 1.47) (high education) < 100 vs. >100 m
[38]	ALSWH	Australia	2006–2011	Cross sectional	269,912 (100%)	Range: 31–90	Doctor-diagnosed	Age, smoking, behavior, BMI, area	Prevalence
									NO_2_ 1.04 (0.91, 1.20) per 3.7 ppb^c^
									Distance: 0.99 (0.95, 1.04) 3 per 1 km
[64]	CAFEH	Boston, Massachusetts, United States	2009–2012	Cross sectional	653 (58%)	60	Doctor-diagnosed	Age, iSES	PrevalencePNC 0.71 (0.46, 1.10) per 1 particles/cm^3^; log-transformed
[65]	CHAMPIONS	Leicestershire, United Kingdom	2004–2011	Cross sectional	10,443 (47%)	59	Clinical examination	Age, sex, iSES, nSES, smoking, behavior, BMI, area	Prevalence
									NO_2_ 1.10 (0.92, 1.32) per 10 μg/m^3^ ^c^
									PM_10_ 1.3 (0.5, 2.9) per 10 μg/m^3^ ^c^
									PM_2.5_ 1.6 (0.4, 4.6) per 10 μg/m^3^ ^c^
[66]	MESA	Multiple cities, United States	2000–2012	Cohort	5,135 (53%)	62–64 (with diabetes)	Clinical examination	Age, sex. iSES, nSES, smoking, behavior, BMI, familial diabetes, area	Incidence
									NO_x_ 1.04 (0.77, 1.40) per 47.1 ppb^a^
									PM_2.5_ 1.05 (0.87, 1.26) per 2.43 μg/m^3^ ^a^
									Distance 0.96 (0.80, 1.16) <100 vs. >100 m
									Prevalence
									NO_x_ 1.29 (0.94, 1.76) per 47.1 ppb
									PM_2.5_ 1.16 (0.94, 1.42) per 2.43 μg/m^3^ ^c^
									Distance 1.10 (0.91, 1.34) <100 vs. >100 m
[67]	Nurses’ Health Health Professionals Follow-Up	United States	1989–2002	Cohort	89,460 (83%)	55	Multimodal	Age, sex, iSES, smoking, behavior, BMI, familial diabetes, hypertension, year, area	IncidenceDistance 1.11 (1.01, 1.23) 0–49 vs. >200 mDistance 0.96 (0.63, 1.48) 50–99 vs. >200 mDistance 0.96 (0.87, 1.06) 100–199 vs. >200 m
[32]	Rome Longitudinal	Rome, Italy	2008–2013	Cohort	1,319,193 (55%)	Range: 35–70	Administrative data from hospital and insurance registries	Age, sex, iSES	Incidence
									NO_2_ 1.00 (1.00, 1.01) per 10 μg/m^3^ ^c^
									NO_x_ 1.01 (1.00, 1.01) per 20 μg/m^3^ ^c^
									PM_2.5abs_ 1.00 (0.98, 1.02) per 1 × 10^−5^/m^c^
									PM_10_ 1.00 (0.99, 1.02) per 10 μg/m^3^
									PM_2.5_ 1.00 (0.98, 1.02) per 5 μg/m^3^ ^c^
									PMcoarse 0.99 (0.97, 1.02) per 10 μg/m^3^
									Prevalence
									NO_2_ 1.00 (1.00, 1.01) per 10 μg/m^3^ ^c^
									NO_x_ 1.01 (1.00, 1.01) per 20 μg/m^3^
									PM_2.5abs_ 0.98 (0.96, 0.99) per 1 × 10^−5^/m
									PM_10_ 0.99 (0.98, 1.00) per 10 μg/m^3^ ^c^
									PM_2.5_ 0.98 (0.96, 1.00) per 5 μg/m^3^ ^c^
									PM_coarse_ 0.96 (0.94, 0.98) per 10 μg/m^3^
[68]	ELISABET	Lille and Dunkirk, France	2011–2013	Cross sectional	2,797 (53%)	53	Clinical examination	Age, sex, iSES, smoking, behavior, BMI, area	Prevalence
									NO_2_ 1.06 (0.90, 1.25) per 5 μg/m^3^ ^c^PM_10_ 1.04 (0.86, 1.25) per 2 μg/m^3^ ^c^
[39]	HNR	Ruhr Areas, Germany	2000–2008	Cohort	3,607 (52%)	59	Clinical examination	Age, sex, iSES, nSES, smoking, behavior, BMI, area	Incidence
									PM_10_ 1.05 (1.00, 1.10) per 1 μg/m^3^
									PM_2.5_ 1.03 (0.95, 1.12) per 1 μg/m^3^ ^c^ traffic PM_2.5_ 1.36 (0.97, 1.89) per 1 μg/m^3^
									Distance 1.37 (1.04, 1.81) <100 vs. 100–200 m
[69]	33 CCHS	Multiple cities, China	2009–2009	Cross sectional	15,477 (47%)	45	Clinical examination	Age, sex, iSES, smoking, behavior, BMI, familial diabetes, area	PrevalenceNO_2_ 1.22 (1.12, 1.33) per 9 μg/m^3^
[43]	33 CCHS	Multiple cities, China	2009–2009	Cross sectional	15,477 (47%)	45, both	Clinical examination	Age, sex, iSES, nSES, smoking, behavior, (BMI)^e^, familial CVD, co-pollutants	PrevalenceNO_2_ 1.20 (1.08, 1.32) per 10 μg/m^3^ ^c^
	

Abbreviations: CI, confidence interval; iSES, measures of individual socioeconomic status such as education; income; nSES, measures of neighborhood socioeconomic status such as neighborhood household income; BMI, body mass index; area, area level adjustments such as city DDCH.

^a^ Effect estimates can be ORs, RRs, HRs, or IRRs, depending on the analysis.

^b^ Adjusted for other behavioral factors other than smoking such as diet, alcohol consumption or physical activity.

^c^ Effect estimates included in meta-analysis.

^d^ Adjusted for hypertension, COPD, asthma, congestive heart failure, acute myocardial infarction, and cancer.

^e^ Multimodal strategies to identify diabetes cases include a combination of self-reported doctor-diagnosed cases, clinical examinations of blood sugar levels or use of medication for glycaemic control.

^f^ BMI was not included but considered.

### Study Description

All studies were published after 2010. Nine studies estimated the association of TRAP with incidence of diabetes, 10 with diabetes prevalence, and two with both incidence and prevalence (the Rome Longitudinal [32] and the SAPALDIA study [33, 34]). The majority of the studies were conducted in Europe (10) or North America (8), followed by China (2) and Australia (1). Three studies were exclusively of women (BWHS [35, 36], SALIA [37], ALSWH [38]). NO_2_ or NO_x_ were the most commonly studied pollutants (17), 11 studies investigated at least one particle metric, and seven included proximity metrics. Exposure levels ranged from very low (e.g., Australia, Canada) to high (e.g., Rome, Italy, China), with ranges in annual means of 5–42 µg NO_2_/m^3^ and 4–25 µg PM_2.5_/m^3^. The 11 cohort studies, all conducted in Europe or North America, included 2,931 to over 1 million participants with a range of follow-up of 4–16 years. The ten cross-sectional studies had 513 up to 2.5 million participants.

Diabetes definitions varied, and included self-report of physician-diagnosed diabetes (five studies), disease registers (two studies), administrative data (e.g., insurance claims) indicating diabetes diagnosis or prescription of hypoglycemic medications (three studies), clinical examinations at study centers, measuring blood glucose (five studies), or using a combination of different data sources (blood glucose measurements, questionnaire, medication, data linkage, six studies). Most smaller cohort studies (n < 10,000 participants) used clinical examinations (SAPALDIA, HNR, MESA, CHAMPIONS) or self-reported physician-diagnosed diabetes, whereas larger administrative cohort or cross-sectional studies typically relied on linkage to administrative databases or registers (e.g., ONPHEC, Rome longitudinal, Table 1).

### Results of Meta-Analysis

**FIGURE 1:**
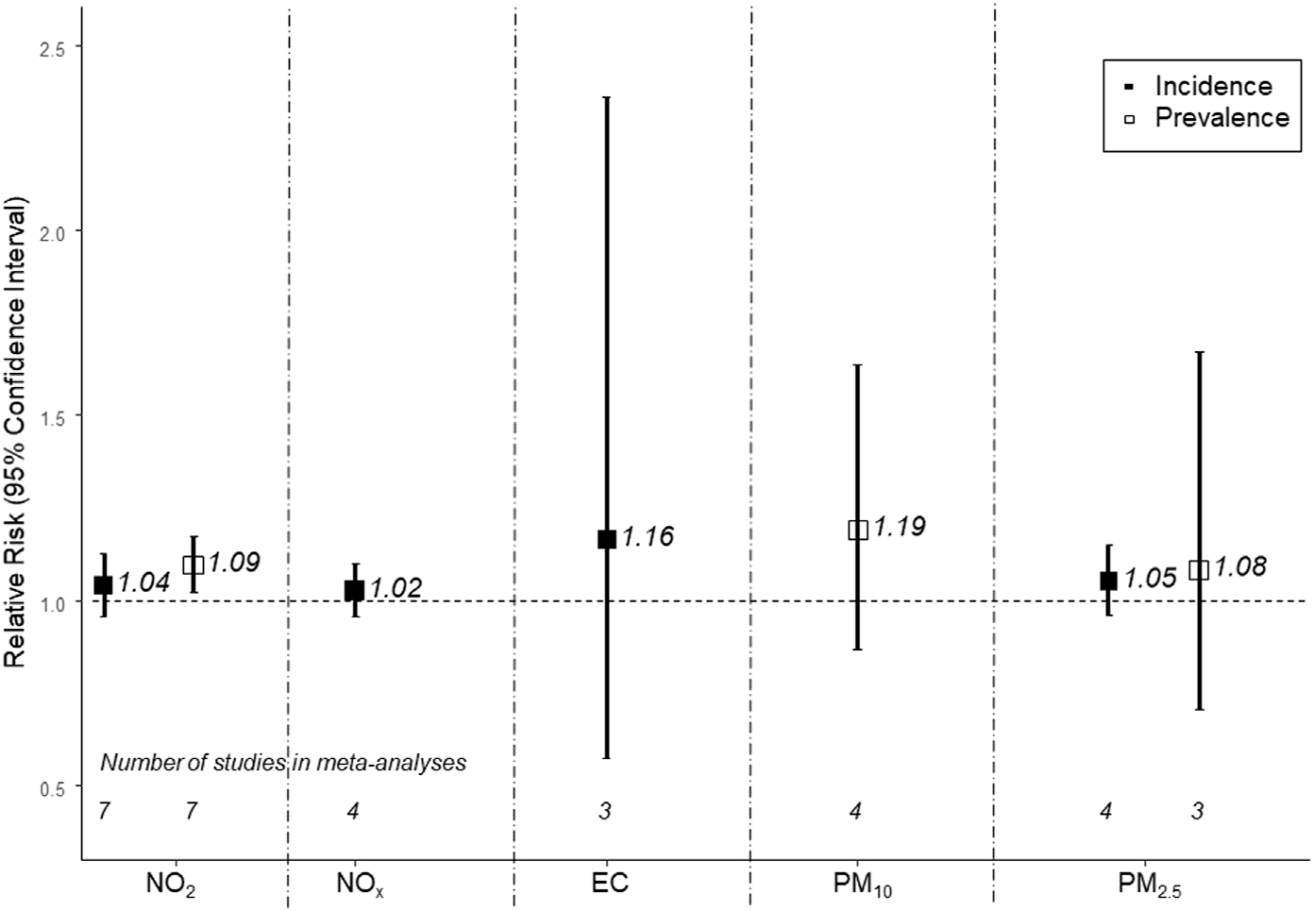
Meta-analysis of associations between traffic-related air pollutants and diabetes prevalence (empty squares) and incidence (filled squares) (Global 2022). The following increments were used: 10 µg/m^3^ for NO_2_, 20 μg/m^3^ for NO_x_, 1 μg/m^3^ for EC, 10 μg/m^3^ for PM_10_, and 5 μg/m^3^ for PM_2.5_. Effect estimates cannot be directly compared across the different traffic-related pollutants because the selected increments do not necessarily represent the same contrast in exposure.

**FIGURE 2:**
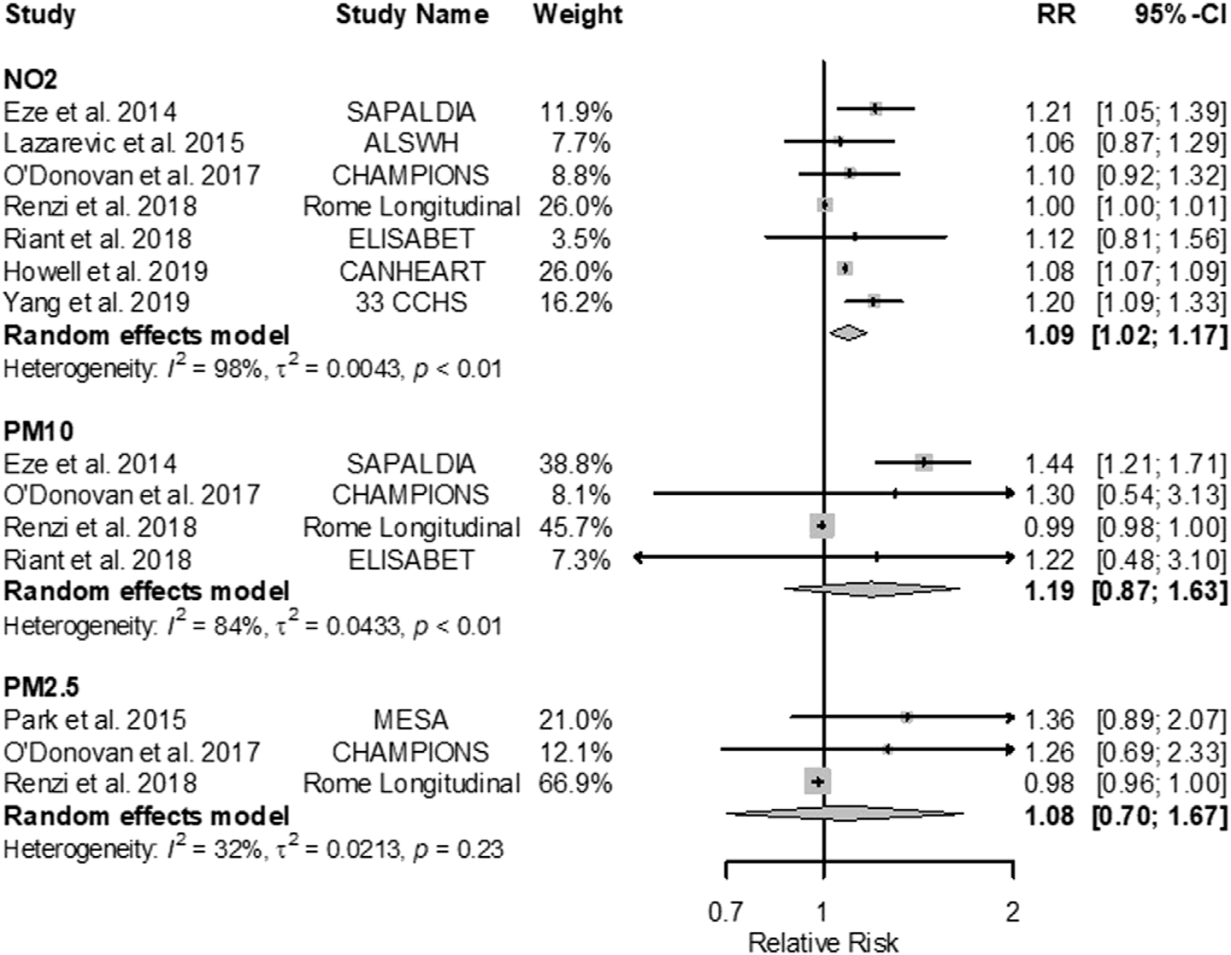
Forest plots of adjusted RRs (95% CIs) for diabetes prevalence with NO_2_, PM_10_, and PM_2.5_ (Global 2022). The size of the grey squares represents the weight of the study in the meta-analysis. The following increments were used: 10 μg/m^3^ for NO_2_, 20 μg/m^3^ for NO_x_, 1 μg/m^3^ for EC, 10 μg/m^3^ for PM_10_, and 5 μg/m^3^ for PM_2.5_. Effect estimates cannot be directly compared across the different traffic-related pollutants because the selected increments do not necessarily represent the same contrast in exposure.

**FIGURE 3:**
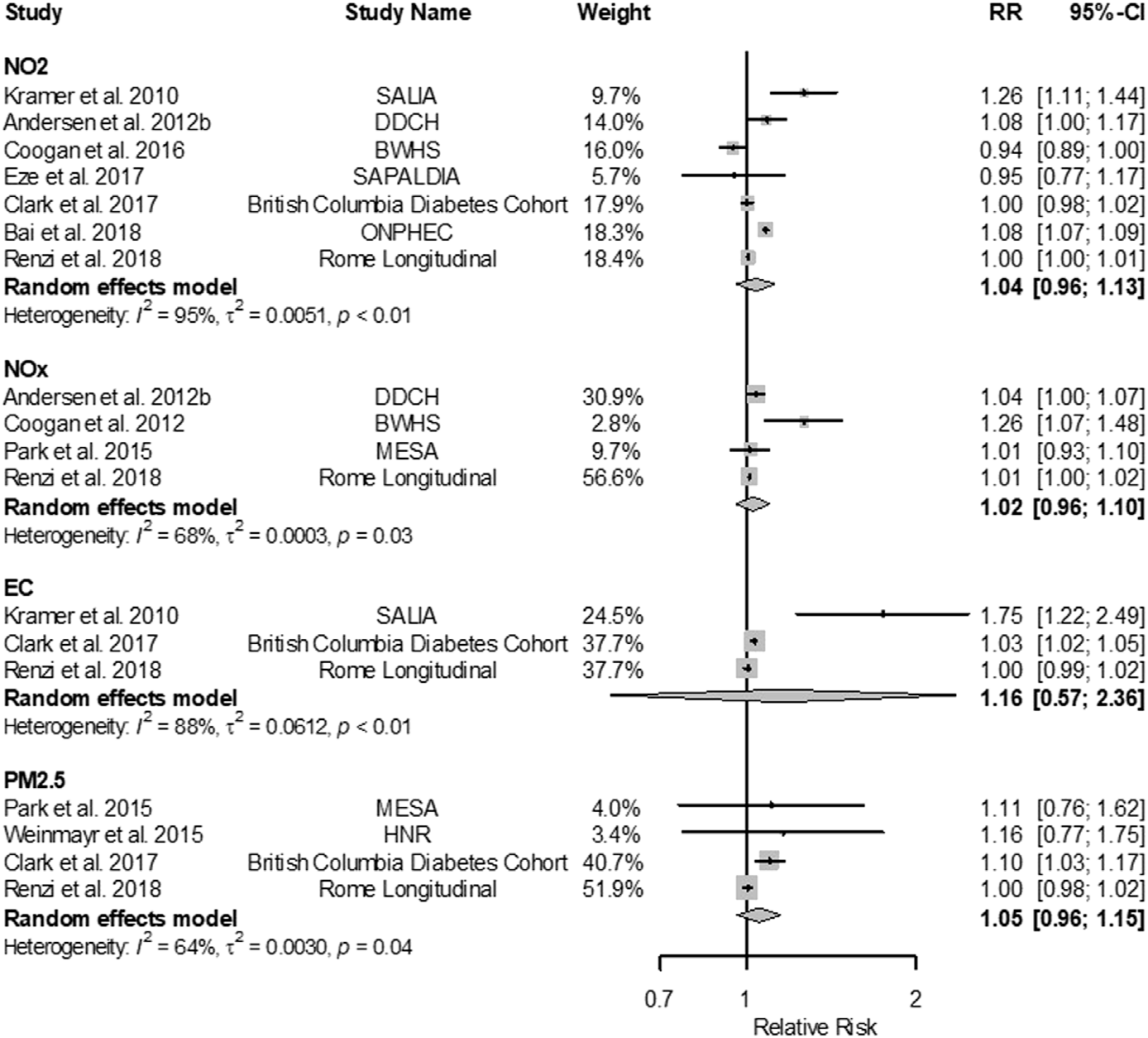
Forest plots of adjusted RRs (95% CIs) for diabetes incidence with NO_2_, NO_x_, EC and PM_2.5_ (Global 2022). The size of the grey squares represents the weight of the study in the meta-analysis. The following increments were used: 10 µg/m^3^ for NO_2_, 20 µg/m^3^ for NO_x_, 1 µg/m^3^ for EC, 10 µg/m^3^ for PM_10_, and 5 µg/m^3^ for PM_2.5_. Effect estimates cannot be directly compared across the different traffic-related pollutants because the selected increments do not necessarily represent the same contrast in exposure.

Meta-analyses indicated positive associations of all traffic-related air pollutants with diabetes incidence and prevalence, though estimates were imprecise (Figure 1). For example, higher exposure to NO_2_, the TRAP for which there were the most studies (seven studies), corresponded to higher diabetes prevalence (RR 1.09; 95% CI: 1.02; 1.17 per 10 μg/m^3^); the individual estimates were highly heterogeneous, especially for the NO_2_ results (Figure 2). The association was less pronounced for diabetes incidence (RR 1.04; 95% CI: 0.96; 1.13 per 10 μg/m^3^; Figure 3). The summary estimates for EC, PM_2.5_ and PM_10_ were also positive but even less precise and based on fewer individual studies.

### Results From Studies Not Entering Meta-Analysis

For pollutants not included in the meta-analyses (such as ultrafine particles PNC or NO, marked in Table 1 without ^c^) elevated risks were observed for measures of NO_x_ but not the various measures of PM in the prevalence analyses. The incidence analyses showed elevated risks for diabetes with NO and PNC. Notably, the traffic-specific PM_2.5_ in the HNR cohort [39] yielded a substantially larger association compared to the total PM_2.5_ mass estimates (RR 1.36 vs. 1.03 or 1.05 per 1 μg/m^3^). All but one study (MESA) showed positive (though imprecise) associations with distance and density of traffic (Table 1, Supplementary Figures S3, S4).

### Risk of Bias and Subgroup and Sensitivity Analysis

The ONPHEC [40], British Columbia Diabetes Cohort [41], CANHEART [42], and Rome Longitudinal study [32] were considered to have high RoB due to incomplete confounder control (missing adjustment for smoking or socioeconomic status). The SAPALDIA cohort [33, 34] was considered to have high potential for selection bias due to long survival in a cohort before inclusion into the analysis and the 33 CCHS study had extensive missing data [43] (Supplementary Table S5).

In subgroup analyses excluding these studies, association magnitudes were similar or larger (Supplementary Tables S6, S7). For example, restricting to prevalence studies with smoking adjustment eliminated heterogeneity entirely and yielded meta-analytic estimates for NO_2_ of 1.09 [95% CI: 1.02; 1.17] (from 1.17 [1.09; 1.25]), and for PM_10_ of 1.19 [0.87; 1.63] (from 1.43 [1.28; 1.59]).

Five studies evaluated confounding by concurrent noise exposure (British Columbia Diabetes Cohort, Plovdiv Diabetes Survey, both SAPALDIA analyses, Rome longitudinal [32–34, 41, 44], Supplementary Table S8). Most TRAP effect estimates were attenuated upon noise adjustment, but still showed elevated risks. For example, the NO_2_ prevalence results in the SAPALDIA study were reduced from 1.21 [1.05; 1.39] to 1.19 [1.03, 1.38] when adjusting for noise [34].

### Confidence Assessments

The modified OHAT assessment was conducted for the 16 studies entering meta-analyses (Table 2). Among factors reducing the quality of the evidence, the most common factor was imprecision (wide CI and including unity despite sufficient sample size). For NO_2_ and diabetes incidence, the confidence was upgraded due to monotonic exposure-response functions reported in two studies [40, 45]. We upgraded the evidence on NO_2_ and prevalence due to potential downward bias. We arrived at a moderate confidence assessment for overall TRAP based on the moderate confidence for NO_2_. While the confidence was low for the other pollutants, the associations for these pollutants were suggestive of an association, though imprecise.

**TABLE 2:** Confidence rating for the quality in the body of evidence for traffic-related air pollution and diabetes (Global 2022).

Pollutant	High ++++		Factors decreasing confidence “0” if no concern; if serious concern to downgrade confidence				Factors increasing confidence “0” if not present; “+” if sufficient to upgrade confidence			Final confidence rating	Rating across study designs
	Moderate +++										
	Low ++										
	Very low +										
	Study design	Initial confidence rating (# studies)	Risk of bias	Unexplained inconsistency	Imprecision	Publication bias	Monotonic exposure-response	Consideration of residual confounding	Consistency across populations		
NO_2_	Cohort	+++ (N = 7)	0	-	-	0	+	0	0	++ (Low)	+++ (Moderate)
	Rationale	Cohort design initially rated as moderate	Four studies with high RoB but results not sensitive to exclusions of those studies	High heterogeneity (I^2^ = 95%), due to both magnitude and direction	Sample size met, but confidence interval wide and includes unity	No formal evaluation possible	Two influential studies show monotonic ERF (Andersen, 2012b; Bai, 2018)	Confounding in both directions possible	Too few studies to evaluate		The combined rating is based on the higher confidence rating. Both study designs show evidence of a positive association, therefore no reason for a downgrade
	Cross-sectional	++ (N = 7)	0	0	0	0	0	+	0	+++ (Moderate)	
	Rationale	Cross-sectional design initially rated as low	Three studies with high RoB, increased or stable effect estimates after excluding high RoB studies	High heterogeneity (I^2^ = 98%) due to magnitude not direction	Sample size met, and confidence interval does not include unity	No formal evaluation possible	No evidence of plausible shape of ERF.	Larger estimates in studies with better confounder control suggests residual confounding toward the null	Across different populations robust effect, but too few studies		
NO_X_	Cohort	+++ (N = 4)	0	0	-	0	0	0	0	++ (Low)	NA
	Rationale	Cohort design initially rated as moderate	One study high RoB, but increased estimate after exclusion	Moderate heterogeneity (I^2^ = 68%) mostly due to magnitude not direction	Sample size met, but confidence interval wide and includes unity	No formal evaluation possible	No evidence of plausible shape of ERF	Confounding in both directions possible	Too few studies to assess robustness across populations		
EC	Cohort	+++ (N = 3)	0	0	-	0	0	0	0	++ (Low)	NA
	Rationale	Cohort design initially rated as moderate	Elevated estimate based on one study with moderate RoB. Two studies with high RoB show effect closer to the null	High heterogeneity (I^2^ = 88%) due to magnitude not direction	Sample size met, but confidence interval wide and includes unity	No formal evaluation possible	No evidence of plausible shape of ERF.	Confounding in both directions possible	Insufficient evidence for robustness across populations		
PM_10_	Cross-sectional	++ (N = 4)	0	0	-	0	0	0	0	+ (Very low)	NA
	Rationale	Cross-sectional design initially rated as low	One of 4 studies high RoB but increased estimate upon exclusion of the high RoB study	High heterogeneity (I^2^ = 84%) due to magnitude not direction	Sample size met, but confidence interval wide and includes unity	No formal evaluation possible	No evidence of plausible shape of ERF.	Larger estimates in studies with better confounder control, but number of studies considered too small for upgrade	All studies European, no consistency check possible		
PM_2.5_	Cohort	+++ (N = 4)	0	0	-	0	0	0	0	++ (Low)	++ (Low)
	Rationale	Cohort design initially rated as moderate	Two studies high RoB, but increased estimate upon exclusion of high RoB studies	Moderate heterogeneity (I^2^ = 64%) due to magnitude not direction	Sample size met, but confidence interval wide and includes unity	No formal evaluation possible	No evidence of plausible shape of ERF.	Larger estimates in studies with better confounder control, but number of studies considered too small for upgrade	Insufficient evidence for robustness across populations		Both study designs show estimates in the same direction
	Cross-sectional	++ (N = 3)	0	0	-	0	0	0	0	+ (Very low)	
	Rationale	Cross-sectional design initially rated as low	One study high RoB, no sensitivity analysis due to low numbers	Low heterogeneity (I^2^ = 32%)	Sample size met, but confidence interval wide and includes unity	No formal evaluation possible	No evidence of plausible shape of ERF	Larger estimates in studies with better confounder control, but number of studies too small	Insufficient evidence for robustness across populations		

The downgrading factor indirectness and the upgrading factor large magnitude of effect were not considered further.

A confidence rating of moderate was also reached in the narrative assessment that considered all studies. This rating was based on the meta-analytical evidence of an association of NO_2_ with diabetes prevalence and suggestive evidence of an association of NO_2_, NO_x_, traffic-related PM with incident and prevalent diabetes. The confidence in the evidence was further supported by the monotonic exposure-response relationships reported in two studies, positive albeit imprecise associations involving indirect traffic measures, and numerous positive associations from studies that adjusted for likely confounders. Further, associations generally remained positive after adjustment for noise exposure (Supplementary Table S8). Finally, effect estimates were larger among the subgroup of studies with more extensive confounder adjustment, and among studies that used comprehensive outcome ascertainment methods (versus self-report and administrative data) (Supplementary Tables S6, S7).

### Study Characteristic and Supplemental Analysis of Studies From the Extended Search

Since our systematic search ending in July 2019, new studies have been published on TRAP and diabetes. We extended our search to May 2022 resulting in 304 hits. Five studies met the inclusion criteria (Table 3) adding estimates to all meta-analyses on diabetes incidence and the PM_2.5_ prevalence analyses (Supplementary Figures S5–S7). While the pooled estimates did not change dramatically, risk estimates were still elevated and confidence intervals became narrower; especially for the PM_2.5_-incidence analyses that was borderline significant (Supplementary Figure S5). Additionally, the Danish study [46] with traffic-specific pollutant estimates and the HNR analysis from 2020 [47] with longer follow-up and refined source-specific exposure assessment as compared to the 2015 analysis [39] showed significantly elevated risks related to traffic-specific NO_2_, EC, and PM_2.5_. Both also add to the evidence on ultrafine particles. However, measures were not comparable and thus meta-analysis was not possible for the different metrics of UFP. Overall, the results of the HEI 2022 review were strengthened by supplemental analyses of the studies identified in the updated search.

**TABLE 3:** Characteristics of the studies from extended search up to May 2022 reporting on the association of traffic-related air pollution and diabetes incidence or prevalence (Global 2023).

Reference	Study name	Location	Study period	Study design in analysis	Sample size N (% women)	Age at baseline	Ascertainment of diabetes	Confounder adjusted for	Results (estimate^a^, 95% CI, increment)
[47]	HNR	Ruhr Areas, Germany	2006–2015	Cohort	2,451 (52%)	58	Self-reported or medication or clinical examination	Age, sex, smoking, behavior, noise (extended models unchanged results iSES, nSES)	Incidence
									NO_2_: 1.02 (0.99, 1.05) per 1 μg/m^3^ ^b^ traffic NO_2_: 1.06 (1.01, 1.12) per 1 μg/m^3^
									PM_10_: 1.06 (1.01, 1.12) per 1 μg/m^3^ traffic PM_10_: 2.00 (1.19, 3.34) per 1 μg/m^3^
									PM_2.5_: 1.06 (0.98, 1.16) per 1 μg/m^3^ ^b^ traffic PM_2.5_: 2.13 (1.26, 3.61) per 1 μg/m^3^
									PNC<1: 1.29 (1.10, 1.53) per 500 particles/mL traffic PNC < 1: 2.11 (1.04, 4.28) per 500 particles/mL
[46]	National Danish Register	Denmark	2005–2017	Prospective cohort	2,631,488 (51.4%)	52	Administrative data from hospital and prescription registers	Age, sex, iSES, nSES	Incidence
									NO_2_: 1.056 (1.046, 1.065) per 7.15 μg/m^3^ ^b^ traffic NO_2_: 1.039 (1.031, 1.047) per 5.17 μg/m^3^
									EC: 1.022 (1.016, 1.027) per 0.28 μg/m^3^ ^b^ traffic EC: 1.037 (1.030, 1.043) per 0.17 μg/m^3^
									PM_2.5_: 1.043 (1.031, 1.056) per 1.85 μg/m^3^ ^b^ traffic PM_2.5_: 1.026 (1.020, 1.031) per 0.37 μg/m^3^
									PNC: 1.052 (1.042, 1.063) per 4,248 particles/mL traffic PNC: 1.049 (1.040, 1.058) per 1,698 particles/mL
[70]	487 Municipalities	Multiple cities, Indonesia	2013	Cross sectional	647,947 (52%)	42	Self-reported	Age, sex, iSES, smoking, behavior, BMI, area, intermediate	PrevalencePM_2.5_: 1.09 (1.05, 1.14) per 10 μg/m^3^
[71]	JHS	Jackson, Mississippi, United States	2000–2008	Cohort	5,128 (63%)	55	Clinical examination or medication	Age, sex, nSES, smoking, behavior, familial diabetes, BMI, others, area	Incidence
									PM_2.5_: 1.09 (0.90, 1.32) per 0.81 μg/m^3^ ^b^
									Prevalence
									PM_2.5_: 1.08 (1.00, 1.17) per 0.81 μg/m^3^
									Distance: 0.91 (0.61, 1.36) <150 vs. 1,000 m
									Distance: 0.94 (0.74, 1.20) 150–299 vs. 1,000 m
									Distance: 1.01 (0.91, 1.12) 300–999 vs. 1,000 m
[72]	SALSA	Sacramento, California, United States	1998–2007	Cohort	1,075 (59%)	71	Self-reported, medication or clinical examination	Age, sex, iSES, nSES, smoking, co-pollutant	Incidence
									NO_2_: 1.02 (0.98, 1.05) per 6.1 ppb^b^
									NO_x_: 1.13 (0.96, 1.33) per 2.3 ppb^b^
									PM_2.5_: 1.20 (1.03, 1.40) per 1.9 μg/m^3^ ^b^

Abbreviations: CI, confidence interval; iSES, measures of individual socioeconomic status such as education; income; nSES, measures of neighborhood socioeconomic status such as neighborhood household income, BMI, body mass index; area, area level adjustments such as city DDCH.

^a^ Effect estimates can be ORs, RRs, HRs, or IRRs, depending on the analysis.

^b^ Effect estimates included in meta-analysis

## Discussion

In this comprehensive systematic review of epidemiologic evidence on the association of TRAP with adult diabetes, we identified 21 pertinent studies. Our summary estimates generally suggested an adverse association of TRAP with diabetes risk, although some of the effect estimates were imprecise and based on small numbers of studies per pollutant-outcome pair. A statistically significant association was reported between NO_2_ and diabetes prevalence with a summary estimate of 1.09 (95% CI: 1.02; 1.17) per 10 μg/m^3^, supported by consistently positive but imprecise estimates for the other traffic-related air pollutants. Results were strengthened by the reporting of a monotonic exposure-response function in two studies [40, 45], positive associations in studies examining indirect traffic measures, and robust results correcting for traffic noise. The confidence assessment yielded a moderate confidence in the evidence for an association between long-term exposure to TRAP and diabetes. We noted more consistent associations of TRAP with diabetes prevalence than incidence.

The newly identified five studies, with mostly rigorous outcome assessments strengthened the results. Confidence intervals of meta-analytic estimates in the supplemental analyses were less wide, though estimates were still not significantly elevated.

### Findings in Relation to Other Reviews

Recent reviews of ambient air pollution—as opposed to our focus on traffic-related air pollution—in association with diabetes found similar results (Supplementary Table S9). With a larger study base, Lui et al. [6] and Yang et al. [5] not only reported significantly elevated risks for diabetes prevalence with NO_2_, but also with PM_10_, and PM_2.5_ (for example, including 11 studies vs. 3 studies in the PM_2.5_ prevalence analyses). Diabetes incidence risk was significantly elevated with PM_2.5_ in both reviews, and additionally with PM_10_ in the analysis by [5] considering two more studies. As in our analysis, the reviews did not find a significantly elevated risk with NO_2_ and diabetes incidence. Effect estimates seemed slightly larger in our prevalence analysis, though more imprecise (for example, 1.09 [1.02; 1.17] vs. 1.05 [1.03; 1.08] and 1.07 [1.04; 1.11]) in the NO_2_ prevalence analysis. Another review reported elevated diabetes risks in association with living close to major roads [48].

### Biological Mechanisms

Plausible pathways regarding how TRAP could lead to diabetes are discussed in the literature. Important mechanisms include oxidative stress induced inflammation leading to endothelial and mitochondrial dysfunction, resulting in impaired insulin signalling and insulin resistance [10]. Animal studies provide evidence that exposure to high concentrations of traffic particles may be a risk factor in the development of diabetes [49–51]. Studies evaluating mechanistic pathways underlying such metabolic perturbations induced by urban PM and near roadway air pollution have identified possible contributory roles played by inflammation and altered fatty acid metabolism. Indeed, Lucht et al. [47] observed that diabetes incidence in an adult population was mediated by markers of inflammation (adiponectin and C-reactive protein). While our results build on evidence found especially for the association with NO_2_, mechanistic studies on NO_2_ are scarce [52] and NO_2_ could be an indicator for other highly correlated pollutants from the same source. However, a recent study on Witstar rats was able to demonstrate reactive oxygen species formation and mitochondrial and endothelial dysfunction after 3 weeks of repeated high NO_2_ exposure [53]. Epidemiologic studies also found TRAP-associated higher risks for glucose homeostasis dysregulation measured as insulin concentration in cord blood, fasting blood glucose, insulin sensitivity, HOMA-IR, HbA1c in newborns [54], children [55, 56], adolescents [57], and adults [58] indicating a role of early-life exposure.

### Strengths

The systematic approach to study selection and evaluation using an *a priori* specified framework for exposure assessment and for a systematic evaluation of the epidemiological evidence are major strengths of this review. Even though none of the pollutants are uniquely traffic-specific, the use of several indicators of TRAP allowed the evaluation of consistency across pollutants and enabled the Panel to base its conclusions on a larger number of studies with diverse exposure metrics. Additionally, the application of two complementary methods (the modified OHAT assessment for studies entering meta-analyses and the narrative assessment considering all studies for the evaluation of the epidemiological evidence maximizes what can be learned from the epidemiologic studies, including evidence from less studied pollutants like UFP and traffic-specific PM fractions.

### Limitations

The overall number of studies per pollutant was small, limiting our ability to conduct meta-analysis or subgroup analysis for some exposure-outcome pairs, and to investigate publication bias.

It has been proposed that effects of air pollutants on the metabolic system commence at an early age [54, 55]. Studies entering this review, including the newest available studies, comprised older adult populations (mean age >50 years) and have excluded persons with already manifest pollutant-dependent diabetes at baseline from the incidence analyses. Thus, a selection bias toward a healthier population might have compromised the ability to study associations with diabetes incidence. The subgroup analysis showed more robust results for studies with low risk of selection bias (Supplementary Table S6).

Another limitation refers to the possible underestimation and misclassification of diabetes. This may depend on the age of the study participants regarding results on incidence of diabetes or on study design and available data sources. Cohort studies with individual data or smaller cross-sectional studies show more rigorous outcome ascertainment with less risk of bias as opposed to the larger studies based on administrative data. Reliance on self-report or documented disease would miss 24% up to 50% of cases depending on the region, while in-depth study center examinations will have a much higher sensitivity due to the long oligosymptomatic prediagnostic phase of diabetes [2]. Non-differential outcome misclassification (independent from exposure status) related to incomplete case ascertainment might bias the results to the null [59, 60]. This was seen for prevalence studies in the sub-group analysis regarding risk of bias due to outcome ascertainment, but not incidence studies (Supplementary Tables S6, S7).

We were not able to distinguish between type 1 and type 2 diabetes. Since 90% of adult diabetes cases are type 2, and the vast majority of incident diabetes cases in adults are type 2 diabetes, we conclude that our results primarily refer to type 2 diabetes.

### Future Research

In cities, where the majority of the world´s population resides, traffic remains an important source of air pollution. The majority of studies were from high-income countries in Europe and North America with generally lower levels of air pollution than in other world regions. However, the one study from China with mean exposure at the higher end of the exposure range (35.3 μg/m^3^ NO_2_) also showed increased risk of diabetes. The available evidence provides overall moderate evidence that TRAP increase diabetes risk. Large studies with rigorous case ascertainment are needed, including in low and middle income countries and other locations with higher exposures. Studies are also needed to assess the change in composition of TRAP due to diesel and gasoline fleet turnover to lower-emission vehicles with a rising share of non-tailpipe emissions in the overall share of traffic-related particulate matter (e.g., from SO_2_ emissions). The interplay of TRAP with co-exposures in polluted spaces, most notably noise and green space, needs to be better understood for effective intervention [61].

Studies assessing critical windows of exposure, e.g., in younger populations and preclinical outcomes along the mechanistic path to clinically manifest disease are warranted. Evidence suggests that underlying pathology may be underway as early as childhood and adolescence [62]. Future experimental studies should provide more mechanistic evidence for a better understanding of the molecular and cellular actions of long-term exposure to NO_x_ and other TRAP on the cardiometabolic system.

### Conclusion

In conclusion, we found moderate confidence in the evidence for an association of long-term exposure to traffic-related air pollution and diabetes, with higher effect estimates observed in prevalence studies. We observed increased risks in populations in various geographical regions and contexts and conclude, that TRAP is a risk factor for diabetes.

## References

[1] Goyal R Jialal I. Diabetes Mellitus Type 2. StatPearls. Treasure Island (FL): StatPearls Publishing LLC. (2021). Available at: https://www.ncbi.nlm.nih.gov/books/NBK513253/ (Accessed November 12, 2021).

[2] International Diabetes Federation. IDF Diabetes Atlas. Brussels, Belgium: International Diabetes Federation (2021).

[3] Beulens JWJ Pinho MGM Abreu TC den Braver NR Lam TM Huss A Environmental Risk Factors of Type 2 Diabetes-An Exposome Approach. Diabetologia (2021) 65(2):263–74. 10.1007/s00125-021-05618-w 34792619

[4] Wu Y Fu R Lei C Deng Y Lou W Wang L Estimates of Type 2 Diabetes Mellitus Burden Attributable to Particulate Matter Pollution and its 30-Year Change Patterns: A Systematic Analysis of Data from the Global Burden of Disease Study 2019. Front Endocrinol (Lausanne) (2021) 12:689079. 10.3389/fendo.2021.689079 34484113 PMC8414895

[5] Yang BY Fan S Thiering E Seissler J Nowak D Dong GH Ambient Air Pollution and Diabetes: A Systematic Review and Meta-Analysis. Environ Res (2020) 180:108817. 10.1016/j.envres.2019.108817 31627156

[6] Liu F Chen G Huo W Wang C Liu S Li N Associations between Long-Term Exposure to Ambient Air Pollution and Risk of Type 2 Diabetes Mellitus: A Systematic Review and Meta-Analysis. Environ Pollut (2019) 252 (Pt B):1235–45. 10.1016/j.envpol.2019.06.033 31252121

[7] Mozafarian N Hashemipour M Yazdi M Zavareh MHT Hovsepian S Heidarpour M The Association between Exposure to Air Pollution and Type 1 Diabetes Mellitus: A Systematic Review and Meta-Analysis. Adv Biomed Res (2022) 11(1):103. 10.4103/abr.abr_80_21 36660754 PMC9843592

[8] Ren Z Yuan J Luo Y Wang J Li Y. Association of Air Pollution and fine Particulate Matter (PM2.5) Exposure with Gestational Diabetes: a Systematic Review and Meta-Analysis. Ann Transl Med (2023) 11(1):23. 10.21037/atm-22-6306 36760250 PMC9906206

[9] Gorini F Sabatino L Gaggini M Chatzianagnostou K Vassalle C. Oxidative Stress Biomarkers in the Relationship between Type 2 Diabetes and Air Pollution. Antioxidants (Basel) (2021) 10(8):1234. 10.3390/antiox10081234 34439482 PMC8388875

[10] Alderete TL Chen Z Toledo-Corral CM Contreras ZA Kim JS Habre R Ambient and Traffic-Related Air Pollution Exposures as Novel Risk Factors for Metabolic Dysfunction and Type 2 Diabetes. Curr Epidemiol Rep (2018) 5(2):79–91. 10.1007/s40471-018-0140-5 30319933 PMC6178230

[11] Khreis H Nieuwenhuijsen MJ Zietsman J Ramani T. Chapter 1 - Traffic-Related Air Pollution: Emissions, Human Exposures, and Health: An Introduction. In: Khreis H Nieuwenhuijsen M Zietsman J Ramani T, editors. Traffic-Related Air Pollution. Elsevier (2020). p. 1–21. 10.1016/B978-0-12-818122-5.00001-6

[12] Piscitello A Bianco C Casasso A Sethi R. Non-exhaust Traffic Emissions: Sources, Characterization, and Mitigation Measures. Sci Total Environ (2021) 766:144440. 10.1016/j.scitotenv.2020.144440 33421784

[13] HEI. Systematic Review and Meta-Analysis of Selected Health Effects of Long-Term Exposure to Traffic-Related Air Pollution. Boston, MA, USA: Health Effects Institute (2022).

[14] Boogaard H Patton AP Atkinson RW Brook JR Chang HH Crouse DL Long-term Exposure to Traffic-Related Air Pollution and Selected Health Outcomes: A Systematic Review and Meta-Analysis. Environ Int (2022) 164:107262. 10.1016/j.envint.2022.107262 35569389

[15] Higgins J Thomas J Chandler J Cumpston M Li T Page M Cochrane Handbook for Systematic Reviews of Interventions. 2nd ed. Chichester (UK): John Wiley & Sons (2019).

[16] WHO. Risk of Bias Assessment Instrument for Systematic Reviews Informing WHO Global Air Quality Guidelines. Geneva, Switzerland: World Health Organization (2020). Contract No.: WHO/EURO:2020-2669-42425-58853.

[17] Office of Health Assessment Translation. Handbook for Conducting a Literature-Based Health Assessment Using OHAT Approach for Systematic Review and Evidence Integration. Washington, D.C: U.S. Department of Health and Human Services (2019).

[18] HEI (Health Effects Institute). Protocol for a Systematic Review and Meta–Analysis of Selected Health Effects of Long–Term Exposure to Traffic–Related Air Pollution. Massachusetts, United States: Health Effects Institute (2019).

[19] Department for Environment Food & Rural Affairs. Air Quality Library-Defra UK: Department for Environment Food & Rural Affairs (2005). Available from: https://uk-air.defra.gov.uk/library/reports?report_id=306 (Accessed February 22, 2023).

[20] Cyrys J Heinrich J Hoek G Meliefste K Lewné M Gehring U Comparison between Different Traffic-Related Particle Indicators: Elemental Carbon (EC), PM2.5 Mass, and Absorbance. J Expo Anal Environ Epidemiol (2003) 13(2):134–43. 10.1038/sj.jea.7500262 12679793

[21] Janssen NAH Hoek G Simic-Lawson M Fischer P van Bree L ten Brink H Black Carbon as an Additional Indicator of the Adverse Health Effects of Airborne Particles Compared with PM10 and PM2.5. Environ Health Persp (2011) 119(12):1691–9. 10.1289/ehp.1003369 PMC3261976 21810552

[22] Distiller SR. Version 2.29.8 2019 [cited 2021 December 9] (2019). Available from: https://www.evidencepartners.com/ (Accessed February 14, 2019).

[23] WHO. WHO Global Air Quality Guidelines. Particulate Matter (PM2.5 and PM10), Ozone, Nitrogen Dioxide, Sulfur Dioxide and Carbon Monoxide. Geneva: WHO (2021). 34662007

[24] Beelen R Raaschou-Nielsen O Stafoggia M Andersen ZJ Weinmayr G Hoffmann B Effects of Long-Term Exposure to Air Pollution on Natural-Cause Mortality: an Analysis of 22 European Cohorts within the Multicentre ESCAPE Project. Lancet (2014) 383(9919):785–95. 10.1016/S0140-6736(13)62158-3 24332274

[25] Veroniki AA Jackson D Viechtbauer W Bender R Bowden J Knapp G Methods to Estimate the Between-Study Variance and its Uncertainty in Meta-Analysis. Res Synth Methods (2016) 7(1):55–79. 10.1002/jrsm.1164 26332144 PMC4950030

[26] Woodward M. Epidemiology: Study Design and Data Analysis. 3rd ed. Boca Raton, Fla: CRC Press (2014).

[27] Davies HT Crombie IK Tavakoli M. When Can Odds Ratios Mislead? BMJ (1998) 316(7136):989–91. 10.1136/bmj.316.7136.989 9550961 PMC1112884

[28] Khreis H Kelly C Tate J Parslow R Lucas K Nieuwenhuijsen M. Exposure to Traffic-Related Air Pollution and Risk of Development of Childhood Asthma: a Systematic Review and Meta-Analysis. Environ Int (2017) 100:1–31. 10.1016/j.envint.2016.11.012 27881237

[29] Boogaard H Atkinson R Brook J Chang H Hoek G Hoffmann B Evidence Synthesis of Observational Studies in Environmental Health: Lessons Learned from a Systematic Review on Traffic-Related Air Pollution. United States (2023). under review. 10.1289/EHP11532 PMC10664749 37991444

[30] Chen J Hoek G. Long-term Exposure to PM and All-Cause and Cause-specific Mortality: A Systematic Review and Meta-Analysis. Environ Int (2020) 143:105974. 10.1016/j.envint.2020.105974 32703584

[31] Huangfu P Atkinson R. Long-term Exposure to NO(2) and O(3) and All-Cause and Respiratory Mortality: A Systematic Review and Meta-Analysis. Environ Int (2020) 144:105998. 10.1016/j.envint.2020.105998 33032072 PMC7549128

[32] Renzi M Cerza F Gariazzo C Agabiti N Cascini S Di Domenicantonio R Air Pollution and Occurrence of Type 2 Diabetes in a Large Cohort Study. Environ Int (2018) 112:68–76. 10.1016/j.envint.2017.12.007 29253730

[33] Eze IC Foraster M Schaffner E Vienneau D Heritier H Rudzik F Long-term Exposure to Transportation Noise and Air Pollution in Relation to Incident Diabetes in the SAPALDIA Study. Int J Epidemiol (2017) 46(4):1115–25. 10.1093/ije/dyx020 28338949 PMC5837207

[34] Eze IC Schaffner E Fischer E Schikowski T Adam M Imboden M Long-term Air Pollution Exposure and Diabetes in a Population-Based Swiss Cohort. Environ Int (2014) 70:95–105. 10.1016/j.envint.2014.05.014 24912113

[35] Coogan PF White LF Jerrett M Brook RD Su JG Seto E Air Pollution and Incidence of Hypertension and Diabetes Mellitus in Black Women Living in Los Angeles. Circulation (2012) 125(6):767–72. 10.1161/CIRCULATIONAHA.111.052753 22219348 PMC3326581

[36] Coogan PF White LF Yu J Burnett RT Marshall JD Seto E Long Term Exposure to NO2 and Diabetes Incidence in the Black Women's Health Study. Environ Res (2016) 148:360–6. 10.1016/j.envres.2016.04.021 27124624 PMC4874900

[37] Kramer U Herder C Sugiri D Strassburger K Schikowski T Ranft U Traffic-related Air Pollution and Incident Type 2 Diabetes: Results from the SALIA Cohort Study. Environ Health Perspect (2010) 118(9):1273–9. 10.1289/ehp.0901689 20504758 PMC2944089

[38] Lazarevic N Dobson AJ Barnett AG Knibbs LD. Long-term Ambient Air Pollution Exposure and Self-Reported Morbidity in the Australian Longitudinal Study on Women's Health: a Cross-Sectional Study. BMJ Open (2015) 5(10):e008714. 10.1136/bmjopen-2015-008714 PMC4636641 26503387

[39] Weinmayr G Hennig F Fuks K Nonnemacher M Jakobs H Mohlenkamp S Long-term Exposure to fine Particulate Matter and Incidence of Type 2 Diabetes Mellitus in a Cohort Study: Effects of Total and Traffic-specific Air Pollution. Environ Health (2015) 14:53. 10.1186/s12940-015-0031-x 26087770 PMC4479324

[40] Bai L Chen H Hatzopoulou M Jerrett M Kwong JC Burnett RT Exposure to Ambient Ultrafine Particles and Nitrogen Dioxide and Incident Hypertension and Diabetes. Epidemiology (2018) 29(3):323–32. 10.1097/EDE.0000000000000798 29319630

[41] Clark C Sbihi H Tamburic L Brauer M Frank LD Davies HW. Association of Long-Term Exposure to Transportation Noise and Traffic-Related Air Pollution with the Incidence of Diabetes: A Prospective Cohort Study. Environ Health Perspect (2017) 125(8):087025. 10.1289/EHP1279 28934721 PMC5783665

[42] Howell NA Tu JV Moineddin R Chen H Chu A Hystad P Interaction between Neighborhood Walkability and Traffic-Related Air Pollution on Hypertension and Diabetes: The CANHEART Cohort. Environ Int (2019) 132:104799. 10.1016/j.envint.2019.04.070 31253484

[43] Yang BY Guo Y Markevych I Qian ZM Bloom MS Heinrich J Association of Long-Term Exposure to Ambient Air Pollutants with Risk Factors for Cardiovascular Disease in China. JAMA Netw Open (2019) 2(3):e190318. 10.1001/jamanetworkopen.2019.0318 30848806 PMC6484675

[44] Dzhambov AM Dimitrova DD. Exposures to Road Traffic, Noise, and Air Pollution as Risk Factors for Type 2 Diabetes: A Feasibility Study in Bulgaria. Noise Health (2016) 18(82):133–42. 10.4103/1463-1741.181996 27157686 PMC4918667

[45] Andersen ZJ Raaschou-Nielsen O Ketzel M Jensen SS Hvidberg M Loft S Diabetes Incidence and Long-Term Exposure to Air Pollution: a Cohort Study. Diabetes Care (2012) 35(1):92–8. 10.2337/dc11-1155 22074722 PMC3241311

[46] Sørensen M Poulsen AH Hvidtfeldt UA Frohn LM Ketzel M Christensen JH Exposure to Source-specific Air Pollution and Risk for Type 2 Diabetes: a Nationwide Study Covering Denmark. Int J Epidemiol (2022) 51(4):1219–29. 10.1093/ije/dyac040 35285908

[47] Lucht S Hennig F Moebus S Ohlwein S Herder C Kowall B All-source and Source-specific Air Pollution and 10-year Diabetes Incidence: Total Effect and Mediation Analyses in the Heinz Nixdorf Recall Study. Environ Int (2020) 136:105493. 10.1016/j.envint.2020.105493 31991234

[48] Zhao Z Lin F Wang B Cao Y Hou X Wang Y. Residential Proximity to Major Roadways and Risk of Type 2 Diabetes Mellitus: A Meta-Analysis. Int J Environ Res Public Health (2016) 14(1):3. 10.3390/ijerph14010003 28025522 PMC5295254

[49] Chen M Liang S Qin X Zhang L Qiu L Chen S Prenatal Exposure to Diesel Exhaust PM(2.5) Causes Offspring β Cell Dysfunction in Adulthood. Am J Physiol Endocrinol Metab (2018) 315(1):E72–E80. 10.1152/ajpendo.00336.2017 29351483 PMC6087722

[50] Chen M Liang S Zhou H Xu Y Qin X Hu Z Prenatal and Postnatal Mothering by Diesel Exhaust PM(2.5)-exposed Dams Differentially Program Mouse Energy Metabolism. Part Fibre Toxicol (2017) 14(1):3. 10.1186/s12989-017-0183-7 28100227 PMC5423412

[51] Yan YH Chou CC Lee CT Liu JY Cheng TJ. Enhanced Insulin Resistance in Diet-Induced Obese Rats Exposed to fine Particles by Instillation. Inhal Toxicol (2011) 23(9):507–19. 10.3109/08958378.2011.587472 21736501

[52] Forastiere F Peters A. Invited Perspective: The NO2 and Mortality Dilemma Solved? Almost There. Environ Health Perspect (2021) 129(12):121304. 10.1289/EHP10286 34962423 PMC8713649

[53] Karoui A Crochemore C Harouki N Corbiere C Preterre D Vendeville C Nitrogen Dioxide Inhalation Exposures Induce Cardiac Mitochondrial Reactive Oxygen Species Production, Impair Mitochondrial Function and Promote Coronary Endothelial Dysfunction. Int J Environ Res Public Health (2020) 17(15):5526. 10.3390/ijerph17155526 32751709 PMC7432061

[54] Heydari H Najafi ML Akbari A Rezaei H Miri M. Prenatal Exposure to Traffic-Related Air Pollution and Glucose Homeostasis: A Cross-Sectional Study. Environ Res (2021) 201:111504. 10.1016/j.envres.2021.111504 34144009

[55] Toledo-Corral CM Alderete TL Habre R Berhane K Lurmann FW Weigensberg MJ Effects of Air Pollution Exposure on Glucose Metabolism in Los Angeles Minority Children. Pediatr Obes (2018) 13(1):54–62. 10.1111/ijpo.12188 27923100 PMC5722706

[56] Mann JK Lutzker L Holm SM Margolis HG Neophytou AM Eisen EA Traffic-related Air Pollution Is Associated with Glucose Dysregulation, Blood Pressure, and Oxidative Stress in Children. Environ Res (2021) 195:110870. 10.1016/j.envres.2021.110870 33587949 PMC8520413

[57] Thiering E Markevych I Brüske I Fuertes E Kratzsch J Sugiri D Associations of Residential Long-Term Air Pollution Exposures and Satellite-Derived Greenness with Insulin Resistance in German Adolescents. Environ Health Perspect (2016) 124(8):1291–8. 10.1289/ehp.1509967 26863688 PMC4977044

[58] Zhang S Mwiberi S Pickford R Breitner S Huth C Koenig W Longitudinal Associations between Ambient Air Pollution and Insulin Sensitivity: Results from the KORA Cohort Study. Lancet Planet Health (2021) 5(1):e39–e49. 10.1016/S2542-5196(20)30275-8 33421408

[59] Chen Q Galfalvy H Duan N. Effects of Disease Misclassification on Exposure-Disease Association. Am J Public Health (2013) 103(5):e67–73. 10.2105/AJPH.2012.300995 PMC3698812 23488509

[60] Copeland KT Checkoway H McMichael AJ Holbrook RH. Bias Due to Misclassification in the Estimation of Relative Risk. Am J Epidemiol (1977) 105(5):488–95. 10.1093/oxfordjournals.aje.a112408 871121

[61] Rugel EJ Brauer M. Quiet, Clean, green, and Active: A Navigation Guide Systematic Review of the Impacts of Spatially Correlated Urban Exposures on a Range of Physical Health Outcomes. Environ Res (2020) 185:109388. 10.1016/j.envres.2020.109388 32244108

[62] Raghuveer G White DA Hayman LL Woo JG Villafane J Celermajer D Cardiovascular Consequences of Childhood Secondhand Tobacco Smoke Exposure: Prevailing Evidence, Burden, and Racial and Socioeconomic Disparities: A Scientific Statement from the American Heart Association. Circulation (2016) 134(16):e336–e359. 10.1161/CIR.0000000000000443 27619923 PMC5207215

[63] Dijkema MB Mallant SF Gehring U van den Hurk K Alssema M van Strien RT Long-term Exposure to Traffic-Related Air Pollution and Type 2 Diabetes Prevalence in a Cross-Sectional Screening-Study in the Netherlands. Environ Health (2011) 10:76. 10.1186/1476-069X-10-76 21888674 PMC3200985

[64] Li Y Lane KJ Corlin L Patton AP Durant JL Thanikachalam M Association of Long-Term Near-Highway Exposure to Ultrafine Particles with Cardiovascular Diseases, Diabetes and Hypertension. Int J Environ Res Public Health (2017) 14(5):461. 10.3390/ijerph14050461 28445425 PMC5451912

[65] O'Donovan G Chudasama Y Grocock S Leigh R Dalton AM Gray LJ The Association between Air Pollution and Type 2 Diabetes in a Large Cross-Sectional Study in Leicester: The CHAMPIONS Study. Environ Int (2017) 104:41–7. 10.1016/j.envint.2017.03.027 28411585

[66] Park SK Adar SD O'Neill MS Auchincloss AH Szpiro A Bertoni AG Long-term Exposure to Air Pollution and Type 2 Diabetes Mellitus in a Multiethnic Cohort. Am J Epidemiol (2015) 181(5):327–36. 10.1093/aje/kwu280 25693777 PMC4339386

[67] Puett RC Hart JE Schwartz J Hu FB Liese AD Laden F. Are Particulate Matter Exposures Associated with Risk of Type 2 Diabetes? Environ Health Perspect (2011) 119(3):384–9. 10.1289/ehp.1002344 21118784 PMC3060003

[68] Riant M Meirhaeghe A Giovannelli J Occelli F Havet A Cuny D Associations between Long-Term Exposure to Air Pollution, Glycosylated Hemoglobin, Fasting Blood Glucose and Diabetes Mellitus in Northern France. Environ Int (2018) 120:121–9. 10.1016/j.envint.2018.07.034 30077944

[69] Yang BY Qian ZM Li S Chen G Bloom MS Elliott M Ambient Air Pollution in Relation to Diabetes and Glucose-Homoeostasis Markers in China: a Cross-Sectional Study with Findings from the 33 Communities Chinese Health Study. Lancet Planet Health (2018) 2(2):e64–e73. 10.1016/S2542-5196(18)30001-9 29615239

[70] Suryadhi MAH Suryadhi PAR Abudureyimu K Ruma IMW Calliope AS Wirawan DN Exposure to Particulate Matter (PM(2.5)) and Prevalence of Diabetes Mellitus in Indonesia. Environ Int (2020) 140:105603. 10.1016/j.envint.2020.105603 32344253

[71] Weaver AM Bidulescu A Wellenius GA Hickson DA Sims M Vaidyanathan A Associations between Air Pollution Indicators and Prevalent and Incident Diabetes in an African American Cohort, the Jackson Heart Study. Environ Epidemiol (2021) 5(3):e140. 10.1097/EE9.0000000000000140 33912784 PMC8078431

[72] Yu Y Jerrett M Paul KC Su J Shih IF Wu J Ozone Exposure, Outdoor Physical Activity, and Incident Type 2 Diabetes in the SALSA Cohort of Older Mexican Americans. Environ Health Perspect (2021) 129(9):97004. 10.1289/EHP8620 34494856 PMC8425281

