# Supplementary material for: Long-Term Exposure to Traffic-Related Air Pollution and Diabetes: A Systematic Review and Meta-Analysis
Source: Int J Public Health. 2023 May 31;68:1605718. doi: 10.3389/ijph.2023.1605718 (PMC10266340; doi:10.3389/ijph.2023.1605718)
Supplement: Supplementary file 1 [file DataSheet1.docx]

Contents

[List of Abbreviations 1](#_Toc135581203)

[Table S1 Pollutants and metrics considered as TRAP 3](#_Toc135581204)

[Table S2 Exposure framework eligibility criteria matrix 3](#_Toc135581205)

[Table S3 Search terms 4](#_Toc135581206)

[Table S4 List of excluded diabetes studies with reasons (Global 2022 and Global 2023) 10](#_Toc135581207)

[Equation S1 For conversion of effect estimates to a standardized increment of exposure 13](#_Toc135581208)

[Figure S1 Assessing confidence in the quality of the body of evidence following OHAT [42] 13](#_Toc135581209)

[Figure S2 Preferred Reporting Items For Systematic Reviews and Meta-Analysis (PRISMA) flow diagram for the search of the comprehensive review on the association of TRAP with various health outcomes with the focus on diabetes, search up to July 2019. (Global. 2022) 14](#_Toc135581210)

[Figure S3 Forest-plot of the associations between distance measures and diabetes. (Global. 2022) 15](#_Toc135581211)

[Figure S4 Forest-plot of the associations between traffic density measures and diabetes. (Gobal. 2022) 16](#_Toc135581212)

[Table S5 Risk of bias assessment for studies included in meta-analysis: diabetes. (Global. 2022) 17](#_Toc135581213)

[Table S6 Results of the subgroup and sensitivity analysis for the diabetes prevalence. (Global. 2022) 18](#_Toc135581214)

[Table S7 Results of the subgroup and sensitivity analysis for the diabetes incidence. (Global. 2022) 20](#_Toc135581215)

[Table S8 Multi-pollutant analyses in diabetes studies considering noise. (Gobal. 2022) 22](#_Toc135581216)

[Figure S5 Comparison of meta-analytic results of associations between traffic-related air pollutants and diabetes prevalence and incidence from original analyses including studies up to July 2019 (squares) and the updated analysis (triangles) including studies up to May 2022. (Global. 2023) 24](#_Toc135581217)

[Figure S6 Forest plots of adjusted RRs (95% CIs) for diabetes prevalence with NO_2_, PM_10_ and PM_2.5_ from the updated analysis including studies up to May 2022. (Global. 2023) 25](#_Toc135581218)

[Figure S7 Forest plots of adjusted RRs (95% CIs) for diabetes prevalence with NO_2_, NO_x_, EC, and PM_2.5_ from the updated analysis including studies up to May 2022. (Global. 2023) 26](#_Toc135581219)

[Table S9 Comparison of effect estimates with previously published reviews on diabetes prevalence and incidence with ambient air pollution. 27](#_Toc135581220)

[References 28](#_Toc135581221)

# List of Abbreviations

| ALSWH | Australian Longitudinal Study on Women’s Health |
| --- | --- |
| BaP | Benzo(a)pyrene |
| BMI | Body mass index |
| BWHS | Black Women’s Health Study |
| CAFEH | Community Assessment of Freeway Exposure and Health study |
| CANHEART | Cardiovascular Health in Ambulatory Care Research Team |
| 33 CCHS | 33 Communities Chinese Health Study |
| CHAMPIONS | Calculating How Air Pollution Impacts Our Society Study |
| CI | confidence interval |
| COPD | Chronic Obstructive Pulmonary Disease |
| CRS | Concentration response function |
| CVD | Cardiovascular Disease |
| DDCH | Danish Diet, Cancer, and Health cohort |
| EC | elemental carbon, a measure of soot |
| ELISABET | Enquête Littoral Souffle Air Biologie Environnement Study |
| ERF | Exposure risk function |
| GRADE | Grading of Recommendations Assessment, Development and Evaluation (approach) |
| HEI | Health Effects Institute |
| HNR | Heinz Nixdorf Recall study |
| HR | Hazard ratio (or hazard risk) |
| ICD | International classification of disease |
| IDF | International Diabetes Federation |
| IDF | international diabetes federation |
| IRR | Incidence rate ratio |
| iSES | individual socioeconomic status, measures of individual socioeconomic status such as education; income |
| JHS | Jackson Heart Study |
| LUDOK | Literature database on health effects of ambient air pollution |
| MESA | Multi-Ethnic Study of Atherosclerosis |
| MeSH | Medical Subject Headings (keywords set by Pubmed) |
| NA | Not applicable |
| NIEHS OHAT | National Institute of Environmental Health Sciences Office of Health Assessment and Translation |
| NO | nitrous oxide |
| NO_2_ | nitrogen dioxide |
| NO_x_ | nitrogen dioxide and nitrous oxide |
| nSES | neighborhood socioeconomic status, measures of neighborhood socioeconomic status such as mean household income, BMI: body mass index, area |
| OHAT | Office of Health Assessment and Translation |
| ONPHEC | ONtario Population Health and Environment Cohort |
| OR | Odds ratio |
| PAH | Polycyclic aromatic hydrocarbon |
| PECOS | Population, Exposure, Comparator, Outcome and Study |
| PM_10_ | particulate matter with an aerodynamic diameter smaller or equal to 10 micrometer |
| PM_2.5_ | particulate matter with an aerodynamic diameter smaller or equal to 2.5 micrometer |
| PM_2.5abs_ | Light absorption of PM_2.5_, a measure of soot |
| PM_2.5coarse_ | particulate matter with an aerodynamic diameter between 2.5 and 10 micrometer |
| PNC | Particle number count |
| RoB | risk of bias |
| RR | Relative risk or risk ratio |
| SALIA | Study on the influence of Air pollution on Lung function, Inflammation and Ageing |
| SALSA | Sacramento Area Latino Study on Aging |
| SAPALDIA | Swiss cohort study on Air Pollution and Lung Disease In Adults |
| TRAP | traffic-related air pollution |
| UFP | Ultrafine particles, with a diameter of equal to or less than 100nm |
| WHO | World Health Organization |

# Table S1 Pollutants and metrics considered as TRAP

| **Exposure Metric** | **Consideration** |
| --- | --- |
| NO_2_, NO_x_, NO | Frequently used in epidemiological studies; NAAQS or limit values |
| CO | Frequently used particularly in earlier traffic studies; NAAQS or limit values |
| EC, BC, BS, PM absorption (‘soot’)* | Frequently used in epidemiologic studies |
| PM_2.5_, PM_10_, and PM_coarse_ | Frequently used in epidemiological studies; in specific settings PM contrast may have a clearly resolvable relative traffic contribution |
| Non‐tailpipe PM trace metals from wearing of brakes and tires or from the resuspension of road dust, such as Cu, Fe and Zn | Increased interest because of reduction of tailpipe emissions |
| UFP, PNC, quasi‐ultrafine, different particle modes (nucleation, Aitken, accumulation), particle size distribution | Fraction of fine particles produced through combustion and with potentially distinct health effects |
| PAH | Added for completeness; Some increased by traffic, though not a very specific marker and most human exposure is via diet |
| Benzene | Added for completeness; Some VOCs are increased by traffic, though VOCs are generally not specific for traffic. Benzene chosen as a marker for mobile source air toxics |
| Indirect traffic measures (metrics based upon distance or traffic density) | Very specific for local traffic but concerns about validity; indicators represent more than air pollution (e.g., noise) and no quantitative concentration estimates available. |

* Elemental carbon (EC), black carbon (BC), British Smoke (BS), and PM Absorption (PM_abs_) are referred to as EC throughout this report. These carbonaceous pollutants are defined by operational measurement techniques rather than by fundamental chemical properties alone.

# Table S2 Exposure framework eligibility criteria matrix

| **Exposure metric** | **Exposure assessment methods** | **Spatial resolution “pollution surface”** | **Spatial resolution address** | **Spatial resolution address for study identification** | **Traffic contribution to exposure and other considerations^1^** |
| --- | --- | --- | --- | --- | --- |
| All pollutants from Table S2 | Dispersion or CTM models of traffic emissions or traffic-specific source-tracking/apportionment | ≤5 km | ≤5 km | Residential address as exact address, neighborhood, census tract, zip code acceptable (city or county not) | Assumed by method |
| All pollutants from Table S2 | Dispersion or CTM models of all sources | ≤5 km | ≤5 km | Residential address as exact address, neighborhood, census tract, zip code acceptable (city or county not acceptable) | Judgement needed (e.g., required area adjustment in epidemiological analysis if spatial extent of the study area was >10,000 km^2^, determination of whether exposures met long-term criteria) |
| All pollutants from Table S2 | LUR models that contain at least one traffic predictor (e.g., traffic intensity or road density) or broader surrogate of traffic (e.g., address density, household density, population density, impervious surface) | ≤5 km | ≤5 km | Residential address as exact address, neighborhood, census tract, zip code acceptable (city or county not acceptable) | Judgement needed (e.g., required area adjustment if spatial extent of the study area was >10,000 km^2^, determining whether exposures met long-term criteria) |
| PM_2.5_  PM_10_  PM_coarse_ | Surface, satellite and personal monitoring | Excluded | Excluded | Excluded | Excluded |
| Indirect traffic measures (metrics based upon distance or traffic density) | Objective | ≤1000 m from a highway or a major road | ≤100 m | Residential address as exact address or detailed zip code (i.e., street segment) | Assumed by method |

^1^In general, the larger the study area, the less likely a measured or modelled contrast in pollution is primarily due to traffic emissions. Therefore, nationwide epidemiological studies were designated as ‘possibly in’ requiring Panel assessment (see text for additional considerations). The spatial resolution of a pollution surface was selected based on its capacity to identify within-city contrasts in ambient air pollution.

# Table S3 Search terms

The comprehensive search strategy was following the PECOS research questions (Higgins oder Whaley), which can be translated to the diabetes search as: “In the general ADULT population (P), what is the increase in risk of DIABETES (O) for a change (C) in long–term exposure to traffic–related air pollution (E), observed in epidemiologic studies relevant for the health outcome and exposure duration of interest (S)?”

**Search terms for the whole review – diabetes related outcomes highlighted in bold letters in PubMed**

| **PECOS** |  | **Search Terms for Pubmed** |
| --- | --- | --- |
| Population |  | adult[tiab] OR adults[tiab] OR child[tiab] OR children[tiab] OR pupils[tiab] OR preschooler[tiab] OR preschoolers[tiab] OR student[tiab] OR students[tiab] OR adolescent[tiab] OR adolescents[tiab] OR infant[tiab] OR infants[tiab] OR toddler[tiab] OR toddlers[tiab] OR newborn[tiab] OR baby[tiab] OR babies[tiab] OR person[tiab] OR persons[tiab] OR human[tiab] OR humans[tiab] OR people[tiab] OR man[tiab] OR men[tiab] OR woman[tiab] OR women[tiab] OR elderly[tiab] OR boy[tiab] OR boys[tiab] OR girl[tiab] OR girls[tiab] OR patients[tiab] OR population[tiab] OR populations[tiab] OR survivor[tiab] OR survivors[tiab] OR spouse[tiab] OR spouses[tiab] OR wife[tiab] OR husband[tiab] OR smoker[tiab] OR smokers[tiab] OR resident[tiab] OR residents[tiab] OR veteran[tiab] OR mother[tiab] OR mothers[tiab] OR father[tiab] OR fathers[tiab] OR “population based”[tiab] OR “cohort”[tiab] OR (("persons"[Mesh] OR "humans"[Mesh]) NOT (animals[Mesh] NOT humans[Mesh])) |
| Exposure | General Terms to be combined with pollutants | ("Environmental Exposure"[Mesh] OR "Environmental Pollution"[Mesh] OR "Air Pollutants"[Mesh] OR "Air Pollution"[Mesh] OR "air pollution"[tiab] OR "air pollutants"[tiab] OR "polluted atmosphere"[tiab] OR "atmospheric pollution"[tiab] OR "polluted air"[tiab] OR "ambient air"[tiab] OR "Inhalation Exposure/adverse effects"[Mesh] OR "Motor Vehicles"[Mesh] OR "Vehicle Emissions"[Mesh] OR "traffic-related"[tiab]) OR ((traffic OR transport) AND air) |
| NOx  CO  Traffic PM  Non-tailpipe emissions and metals  UFPs  Soot/BC  PAHs  Benzene  Proxy measures for traffic incl OHAT traffic terms | Different Pollutants to be combined with OR | ((("Nitrogen Oxides"[Mesh] OR "Nitrogen dioxide"[tiab] OR "NO2"[tiab] OR "NO(2)"[tiab] OR "NOx"[tiab] OR "NO(x)"[tiab] OR "Nitrogen oxide"[tiab] OR "nitrogen oxides"[tiab]))) OR "oxides of nitrogen"[tiab]  "Carbon Monoxide"[Mesh] OR "carbon monoxide"[tiab]  "Particulate Matter"[Mesh:NoExp] OR "Smog"[Mesh] OR “smog”[tiab] OR "Particle Size"[Mesh] OR "PM10"[tiab] OR PM2.5[tiab] OR PM10-2.5[tiab] OR PM2.5-10[tiab] OR PM1[tiab] OR “fine particulate”[tiab] OR "PM10"[tiab] OR "PM2.5"[tiab] OR "PM10-2.5"[tiab] OR "PM2.5-10"[tiab] OR "PM1"[tiab] OR "PM(10)"[tiab] OR "PM(2.5)"[tiab] OR "PM(10-2.5)"[tiab] OR "PM(2.5-10)"[tiab] OR "PM(1)"[tiab] OR "particulate matter"[tiab] OR "PMcoarse"[tiab] OR "PMcoarse"[tiab]  resuspended dust[tiab] OR re-suspended dust[tiab] OR road dust[tiab] OR brake dust[tiab] OR tire dust[tiab] OR tyre dust[Text Word] OR brake wear[tiab] OR tire wear[tiab] OR tyre wear[tiab] OR road wear[tiab] OR debris dust[tiab] OR fugitive dust[tiab] OR diffuse dust[tiab] OR wear dust[tiab] OR non-exhaust[tiab] OR source apportionment[tiab] OR windblown dust[tiab] OR non-tailpipe[tiab] OR mineral dust[tiab]  (nickel[tiab] OR Ni[tiab] OR Copper[tiab] OR Cu[tiab] OR aluminium[tiab] OR aluminum[tiab] OR Al[tiab] OR zinc[tiab] OR Zn[tiab] OR barium[tiab] OR Ba[tiab] OR iron[tiab] OR Fe[tiab] OR copper[tiab] OR Cu[tiab] OR Antimon[tiab] OR Sb[tiab] OR Tinn[tiab] OR Sn[tiab] OR Zirconium[tiab] OR Zr[tiab] OR "trace metals"[tiab]  AND  ("Particulate Matter"[Mesh:NoExp] OR "Smog"[Mesh] OR “smog”[tiab] OR "Particle Size"[Mesh] OR "PM10"[tiab] OR PM2.5[tiab] OR PM10-2.5[tiab] OR PM2.5-10[tiab] OR PM1[tiab] OR “fine particulate”[tiab] OR "PM10"[tiab] OR "PM2.5"[tiab] OR "PM10-2.5"[tiab] OR "PM2.5-10"[tiab] OR "PM1"[tiab] OR "PM(10)"[tiab] OR "PM(2.5)"[tiab] OR "PM(10-2.5)"[tiab] OR "PM(2.5-10)"[tiab] OR "PM(1)"[tiab] OR "particulate matter"[tiab] OR "PMcoarse"[tiab] OR "PMcoarse"[tiab]))  “submicron“[tiab] OR “surface area“[tiab] OR “ultrafine“[tiab] OR “ultrafine particles“[tiab] OR “ultrafine particle“[tiab] OR “nano particle“[tiab] OR “nano particles“[tiab] OR “nanoparticles“[tiab] OR “nanoparticle“[tiab] OR PM0.1[tiab] OR “PM0.1“[tiab] OR “PM(0.1)“[tiab] OR PM0.25[tiab] OR “PM(0.25)“[tiab] OR “PM0.25“[tiab] OR “quasi-ultrafine“[tiab] OR “quasi ultrafine“[tiab] OR “PNC“[tiab] OR “accumulation mode“[tiab] OR “particle number“[tiab] OR "number of particles"[tiab] OR “aitken mode“[tiab]  "Soot"[Mesh] OR soot[tiab] OR "PM2.5 absorbance"[tiab] OR "PM2.5absorbance"[tiab] OR “PM2.5abs”[tiab] OR "black carbon"[tiab] OR "carbon black"[tiab] OR “organic carbon”[tiab] OR “elemental carbon”[tiab] OR “black smoke”[tiab]  "Polycyclic Aromatic Hydrocarbons"[Mesh:NoExp] OR “polycyclic aromatic hydrocarbons”[tiab] OR PAH[tiab] OR "PAH's"[tiab] OR PAHs[tiab] OR "benzo(a)pyrene"[tiab] OR benzopyrene[tiab]  "benzene"[Mesh] OR benzene[tiab] OR BTEX[tiab]  ((((traffic[tiab]) NOT ("Accidents, Traffic"[Mesh] OR safety[tiab] OR accident[tiab] OR accidents[tiab] OR injur*[tiab] OR collision*[tiab] OR crash*[tiab])) OR "traffic intensity"[tiab] OR "traffic density"[tiab] OR "traffic load"[tiab] OR "traffic count"[tiab] OR "road length"[tiab] OR ((proximity[tiab] OR near[tiab] OR distance[tiab] OR nearest[tiab] OR next[tiab] OR close[tiab] OR closest[tiab]) AND (road*[tiab] OR highway*[tiab] OR freeway*[tiab] OR motorway*[tiab] OR interstate[tiab] OR expressway[tiab])))) OR ((vehicle[tiab] OR vehicles[tiab] OR vehicular[tiab] OR auto[tiab] OR automobile[tiab] OR bus[tiab] OR buses[tiab] OR car[tiab] OR truck[tiab] OR trucker[tiab] OR trucks[tiab] OR engine[tiab] OR transport[tiab] OR traffic[tiab]) AND (emissions[tiab] OR exhaust[tiab] OR fume*[tiab])) |
|  | Measures of effect | “risk”[Mesh] OR “risk”[tiab] OR “risks”[tiab] OR “incidence”[Mesh] OR “incidence”[tiab] OR “incident”[tiab] OR "Prevalence"[Mesh] OR “prevalence”[tiab] OR “prevalent”[tiab] OR "Risk Factors"[Mesh] OR "risk factor"[tiab] OR "Odds Ratio"[Mesh] OR "odds"[tiab] OR “onset”[tiab] OR “associated”[tiab] OR “association”[tiab] OR “cause”[tiab] OR “causes”[tiab] OR “caused”[tiab] OR “develop”[tiab] OR “developed”[tiab] OR “prevent”[tiab] OR “prevents”[tiab] OR “prevented”[tiab] OR “increase”[tiab] OR “increased”[tiab] OR “increases”[tiab] OR “effect”[tiab] OR “effects”[tiab] OR “affect”[tiab] OR “affects”[tiab] OR “affected”[tiab] OR “protective”[tiab] OR “protect”[tiab] OR “protected”[tiab] OR “harm”[tiab] OR “harms”[tiab] OR “harmed”[tiab] OR “harmful”[tiab] OR “hazard”[tiab] OR “hazardous”[tiab] OR "Proportional Hazards Models"[Mesh] OR "proportional hazard"[tiab] |
| **Outcome** | Mortality  Respiratory Effects  Cardiovascular effects  Diabetes  Cancer: Childhood Leukaemia  Birth Outcomes  Pregnancy outcomes  Neuro outcomes  Children  *Additional* search terms for adult outcomes | **Specific Outcomes / Diseases**  ("Mortality"[Mesh] OR "mortality"[MeSH Subheading] OR "Cardiovascular Diseases/mortality"[Mesh] OR "Myocardial Ischemia/mortality"[Mesh] OR "Respiratory Tract Diseases/mortality"[Mesh] OR "Respiratory Tract Infections/mortality"[Mesh] OR "Respiration Disorders/mortality"[Mesh] OR "Lung Neoplasms/mortality"[Mesh] OR "Pulmonary Disease, Chronic Obstructive/mortality"[Mesh]) OR (("cause-specific"[tiab] OR "all-cause"[tiab] OR "non-accidental"[tiab] OR "natural"[tiab] OR "natural-cause"[tiab] OR "cardiovascular"[tiab] OR "respiratory"[tiab] OR "cardiorespiratory"[tiab] OR "cardio respiratory"[tiab] OR "lung cancer"[tiab] OR "COPD"[tiab]) AND (mortality[tiab] OR death[tiab] OR "deadly"[tiab] OR died[tiab] OR fatal*[tiab] OR surviv*[tiab])) OR ("mortality"[tiab] OR "death"[tiab])  "Pulmonary Ventilation"[Mesh] OR "Respiratory Function Tests"[Mesh] OR “spirometry”[tiab] OR "plethysmography"[tiab] OR “forced expiratory”[tiab] OR “FEV”[tiab] OR “FVC”[tiab] OR “FEF25-75”[tiab] OR “MEF”[tiab] OR “expiratory flow”[tiab] OR “expiration flow”[tiab] OR “small airway”[tiab] OR “impulse oscillometry”[tiab] OR “FOT”[tiab] OR “peripheral airway”[tiab] OR (("pulmonary"[tiab] OR "respiratory"[tiab] OR "lung"[tiab]) AND ("volume"[tiab] OR "function"[tiab] OR "ventilation"[tiab] OR "capacity"[tiab]))  OR  "Asthma"[Mesh] OR asthma[tiab] OR asthmatic[tiab] OR wheezing[tiab] OR wheeze[tiab] OR whistle[tiab] OR whistling[tiab] OR "bronchial hyperreactivity"[tiab] OR "Bronchial Hyperreactivity"[Mesh] OR "bronchial hyperresponsiveness"[tiab] OR "airway hyperresponsiveness"[tiab] OR ISAAC[tiab] OR "Respiratory Hypersensitivity/chemically induced"[Mesh] OR bronchiodilat*[tiab] OR "bronchial dilation"[tiab] OR "bronchial dilatation"[tiab] OR bronchioconstrict*[tiab] OR salbutamol*[tiab] OR "methacholine"[tiab] OR "mannitol"[tiab] OR  "Breath Tests"[Mesh] OR “exhaled nitric oxide”[tiab] OR “FeNO”[tiab] OR “fractional exhaled NO”[tiab]  OR  "Acute lower respiratory infection"[tiab] OR "Acute lower respiratory tract infection"[tiab] OR "ALRI"[tiab] OR ("respiration tract"[tiab] AND "infection"[tiab]) OR "Pneumonia"[Mesh] OR "pneumonia"[tiab] OR "Bronchiolitis"[tiab] OR "Bronchitis"[Mesh] OR "Bronchitis"[tiab]  OR  "Pulmonary Disease, Chronic Obstructive"[Mesh] OR COPD[tiab] OR ((“chronic obstructive”[tiab]) AND (bronchitis[tiab] OR “bronchopulmonary disease”[tiab] OR “lung disorder”[tiab] OR “pulmonary disease”[tiab] OR “pulmonary disorder”[tiab] OR “respiratory disease”[tiab] OR disease[tiab])) OR "emphysema"[tiab] OR "chronic airway obstruction"[tiab] OR "chronic airflow obstruction"[tiab]  *This search term includes the general term “cardiorespiratory” which will also be relevant for the mortality studies*  (“cardiovascular”[Title/Abstract] OR “cardiorespiratory”[Title/Abstract] OR “cardio-respiratory”[Title/Abstract]) OR  ("Myocardial Ischemia"[Mesh] OR ((myocardial[tiab] OR myocard[tiab] OR heart[tiab] OR cardiac[tiab] OR cardial[tiab] OR myocardium[tiab]) AND (infarct[tiab] OR infarction[tiab] OR attack[tiab] OR failure[tiab] OR disease[tiab])) OR "Heart Failure"[Mesh] OR “fatal MI”[tiab] OR “coronary event”[tiab] OR “coronary syndrome”[tiab] OR “coronary syndrom”[tiab] OR “cardiac death”[tiab] OR “revascularization”[tiab] OR “revascularisation”[tiab]) OR ("Stroke"[Mesh] OR "Stroke"[tiab] OR "acute cerebrovascular lesion"[tiab] OR "cerebral vasculopathy"[tiab] OR "brain attack"[tiab] OR "cerebral apoplexy"[tiab] OR "brain ischemic attack"[tiab] OR (("cerebrovascular"[tiab] OR "cerebro vascular"[tiab] OR cerebral[tiab]) AND (insufficiency[tiab] OR "accident"[tiab] OR arrest[tiab] OR "failure"[tiab] OR "injury"[tiab] OR "attack"[tiab]))) OR  ("Arteriosclerosis"[Mesh] OR “atherosclerosis”[tiab] OR “arteriosclerosis”[tiab] OR “vascular sclerosis”[tiab] OR "Carotid Intima-Media Thickness"[Mesh] OR “CIMT”[tiab] OR "aorta wall thickness"[tiab] OR "aortic thickness"[tiab] OR "aortic wall thickness"[tiab] OR "arterial thickness"[tiab] OR "artery thickness"[tiab] OR "artery wall thickness"[tiab] OR "carotid intima media thickness"[tiab] OR "carotid intima-media thickness"[tiab] OR "carotid intimamedia thickness"[tiab] OR "intima-media thickness"[tiab] OR "intimal medial thickness"[tiab] OR "intimamedia thickness"[tiab]) OR "Ankle Brachial Index"[Mesh] OR “ankle-brachial index”[tiab] OR "ankle brachial pressure index"[tiab] OR "ankle brachial ratio"[tiab] OR "Pulse Wave Analysis"[Mesh] OR "pulse wave velocity"[tiab] OR "pulse wave analysis"[tiab] OR "augmentation pressure"[tiab] OR "augmentation index"[tiab] OR "vascular reactivity"[tiab] OR "vascular function"[tiab] OR "Vascular Stiffness"[Mesh] OR ((aorta[tiab] OR arterial[tiab] OR aortic[tiab] OR artery[tiab] OR vascular[tiab]) AND (stiffness[tiab] OR stiffening[tiab])) OR "Calcinosis"[Mesh] OR "artery calcification"[tiab] OR "aortic calcification"[tiab] OR ("Blood Pressure"[Mesh] OR “blood pressure”[tiab] OR “systolic pressure”[tiab] OR “diastolic pressure”[tiab] OR "Hypertension"[Mesh] OR “hypertension”[tiab] OR “intravascular pressure”[tiab] OR “vascular pressure”[tiab] OR “blood tension”[tiab] OR “normotension”[tiab] OR “hypertensive”[tiab]) OR  ("Plaque, Atherosclerotic"[Mesh] OR "plaque area"[tiab] OR “atherosclerotic plaque”[tiab] OR “arteriosclerotic plaque”[tiab] OR "atheromatous plaque”[tiab] OR "intima plaque”[tiab])  **"Diabetes Mellitus, Type 2"[Mesh] OR "diabetes"[tiab] OR "diabetic"[tiab] OR T2DM[tiab] OR "type 2 DM"[tiab] OR "fasting blood glucose"[tiab] OR "fasting glucose"[tiab] OR "glucose metabolism"[tiab] OR "glucose homeostasis"[tiab] OR Hba1c[tiab] OR IDDM[tiab] OR NIDDM[tiab] OR HOMA-IR[tiab] OR hyperglycemia[tiab]**  (("Leukemia"[Mesh] OR "Leukemia"[tiab] OR "Leukaemia"[tiab] OR leucemia[tiab] OR leucaemia[tiab] OR "childhood cancer"[tiab] OR hemoblastoma[tiab]) AND ("Child"[Mesh] OR "Adolescent"[Mesh] OR "Young Adult"[Mesh] OR "Infant"[Mesh] OR "children"[tiab] OR "childhood"[tiab] OR child[tiab] OR preschooler[tiab] OR preschoolers[tiab] OR pupil[tiab] OR pupils[tiab] OR student[tiab] OR students[tiab] OR adolescent[tiab] OR adolescents[tiab] OR infant[tiab] OR infants[tiab] OR toddler[tiab] OR toddlers[tiab] OR newborn[tiab] OR newborns[tiab] OR baby[tiab] OR babies[tiab] OR boy[tiab] OR boys[tiab] OR girl[tiab] OR girls[tiab]))  "Fetal Growth Retardation"[Mesh] OR "Birth Weight"[Mesh] OR "Infant, Low Birth Weight"[Mesh] OR "Premature Birth"[Mesh] OR “intrauterine growth restriction”[tiab] OR "Fetal Development"[Mesh] OR “fetal development”[tiab] OR “foetal development”[tiab] OR “intrauterine growth retardation”[tiab] OR "birth weight"[tiab] OR “small for gestational age”[tiab] OR “preterm birth”[tiab] OR “premature birth”[tiab] OR "birth outcome"[tiab] OR "pregnancy outcome"[tiab] OR “neonatal weight”[tiab] OR “newborn weight”[tiab] OR “fetal growth”[tiab] OR “foetal growth”[tiab] OR “foetus growth”[tiab] OR “fetus growth”[tiab] OR “foetal growth restriction”[tiab] OR “foetal growth retardation”[tiab] OR “in utero growth retardation”[tiab] OR “in utero growth restriction”[tiab] OR “congenital hypotrophy”[tiab] OR “prenatal growth retardation”[tiab] OR “prenatal growth restriction”[tiab] OR “retarded intrauterine growth”[tiab] OR “premature childbirth”[tiab] OR “premature birth”[tiab] OR “small for date”[tiab] OR “low birth weight”[tiab] OR (LBW[tiab] AND (infant[tiab] OR baby[tiab] OR newborn[tiab] OR child[tiab])) OR (premature[tiab] AND (infant[tiab] OR baby[tiab] OR newborn[tiab] OR child[tiab])) OR (“preterm”[tiab] AND (infant[tiab] OR baby[tiab] OR newborn[tiab] OR child[tiab]))  "Diabetes, Gestational"[Mesh] OR "Hypertension, Pregnancy-Induced"[Mesh] OR "Gestational Hypertension"[tiab] OR "pregnancy-induced hypertension"[tiab] OR (pregnan*[tiab] AND hypertens*[tiab]) OR pre-eclampsia[tiab] OR preeclampsia[tiab] OR (pregnan*[tiab] AND toxemia*[tiab])  "Cognition Disorders"[Mesh] OR cognition[tiab] OR cognitive[tiab] OR neurobehavio*[tiab] OR neuropsych*[tiab] OR "Mental Processes"[Mesh] OR memory[tiab] OR "mental recall"[tiab] OR (verbal[tiab] OR language[tiab] OR reading[tiab] AND (comprehension[tiab])) OR “language”[tiab] OR learning[tiab] OR perception[tiab] OR perceptual[tiab] OR neurodevelop*[tiab] OR intelligen*[tiab] OR intellect*[tiab] OR “IQ”[tiab] OR behavior[Mesh:NoExp] OR Child behavior[Mesh] OR Adolescent behavior[Mesh] OR Behavioral symptoms[Mesh] OR Spatial behavior[Mesh] OR executive function[tiab] OR “academic achievement”[tiab] OR “academic performance”[tiab] OR  "Neurodevelopmental Disorders"[Mesh] OR attention[tiab] OR inattenti*[tiab] OR hyperactiv*[tiab] OR "impulsive behavior"[Mesh] OR impulsive[tiab] OR impulse-control[tiab] OR impulsivity[tiab] OR “response inhibition”[tiab] OR “inhibitory control”[tiab] OR “vigilance”[tiab] OR “social-behavior”[tiab] OR “social-behaviour”[tiab] OR “social skills”[tiab] OR aggression[tiab] OR aggressive[tiab] OR “ADDH”[tiab] OR “ADHS”[tiab] OR “ADHD”[tiab] OR “ADH”[tiab] OR  "Autism Spectrum Disorder"[Mesh] OR autistic[tiab] OR autism[tiab] OR “Tic-disorder”[tiab] OR Asperger*[tiab] OR “communication-disorder*”[tiab] OR language[tiab] OR agraphia[tiab] OR dyslexi*[tiab] OR dyscalculia[tiab] OR speech[tiab] OR aphasia[tiab] OR echolalia[tiab] OR “stereotyp*”[tiab] OR “Pervasive Developmental Disorder”[tiab] OR “social cognition”[tiab] OR “social communication”[tiab] OR “social reciprocity”[tiab] OR “repetitive behavior*”[tiab] OR “repetitive behaviour”[tiab] OR “restricted interests”[tiab] OR “maladaptive behavior”[tiab] OR “maladaptive behaviour”[tiab] OR “adaptive behavior”[tiab] OR “behavioral regulation”[tiab]  "Aging"[Mesh] OR "Cognitive Dysfunction"[Mesh] OR “dementia”[Mesh] OR dementia[tiab] OR alzheime*[tiab] OR neurotox*[tiab] OR “Neurodegenerative Diseases”[Mesh] OR neurodegenerat*[tiab] OR neurodisease*[tiab] OR Parkinson*[tiab] OR neuropsycholog*[tiab] |
| **Filter** |  | NOT  (((((("shortterm"[ti] OR "short-term"[ti] OR “time series”[ti] OR time-series[ti]) AND (("shortterm"[ti] OR "short-term"[ti] OR “time series”[ti] OR time-series[ti]) NOT ("longterm"[tiab] OR "long term"[tiab] OR "medium term"[tiab] OR "intermediate term"[tiab] OR “chronic”[tiab]))))) OR ("Clinical Trial"[Publication Type] OR "Treatment Outcome"[MeSH] OR "Cross-Over Studies"[Mesh] OR "case cross over"[tiab])) OR ("Air Pollutants, Occupational"[Mesh] OR "Accidents, Traffic"[Mesh] OR "Protective Devices"[Mesh])) OR (mouse[Title/Abstract] OR mice[Title/Abstract] OR rat[Title/Abstract] OR rats[Title/Abstract])  AND  English[Language]  AND  ("1980/01/01"[Date - Publication] : "3000"[Date - Publication]) |
| **Search terms for the LUDOK database** | | |
|  |  | (Sterblichkeit[methods] AND 7L) OR (road[methods] AND 7L) OR (traffic[methods] AND 7L) OR (schwangerschaft[methods] AND 7L) OR (geburt[methods] AND 7L) OR (arteriosklerose[methods] AND 7L) OR (diabetes[methods] AND 7L) OR (leukämie[methods] AND 7L) OR (4O AND 7L) OR (4I AND 7L) OR (4B AND 7L) OR (4E AND 7L) OR (4H AND 7L) OR (4F AND 7L) |

Note 7L is the code for long-term studies, 4O code for outcomes related to pregnancy and prenatal development, 4I outcomes related to outcomes regarding neurocognitive outcomes, 4B = lung function, 4E = acute respiratory outcomes, 4H = cardiovascular outcomes like stroke, blood pressure, 4F = chronic respiratory outcomes, [] indicates the fields searched in the database. the [methods]-field is where LUDOK saves the keywords.

# Table S4 List of excluded diabetes studies with reasons (Global 2022 and Global 2023)

| **Author** | **year** | **Title** | **Reasons for exclusion during full text analysis** |
| --- | --- | --- | --- |
| Requia et al. [5] | 2017 | Association of PM with diabetes, asthma, and high blood pressure incidence in Canada: A spatiotemporal analysis of the impacts of the energy generation and fuel sales | spatial scale too crude (pollution surface), nationwide/statewide study with no or insufficient area-specific adjustments |
| Strak et al. [6] | 2017 | Long-term exposure to particulate matter, NO and the oxidative potential of particulates and diabetes prevalence in a large national health survey | nationwide/statewide study with no or insufficient area-specific adjustments |
| Orioli et al. [7] | 2018 | Association between PM10, PM2.5, NO2, O3 and self-reported diabetes in Italy: A cross-sectional, ecological study | spatial scale too crude (pollution surface) |
| Hazlehurst et al. [8] | 2018 | Individual and Neighborhood Stressors, Air Pollution and Cardiovascular Disease | spatial scale too crude (pollution surface), nationwide/statewide study with no or insufficient area-specific adjustments |
| Bowe et al. [9] | 2018 | The 2016 global and national burden of diabetes mellitus attributable to PM air pollution | nationwide/statewide study with no or insufficient area-specific adjustments |
| Gandini et al. [10] | 2018 | Long term effect of air pollution on incident hospital admissions: Results from the Italian Longitudinal Study within LIFE MED HISS project | spatial scale too crude (pollution surface), nationwide/statewide study with no or insufficient area-specific adjustments |
| Shin et al. [11] | 2019 | Association between long-term exposure of ambient air pollutants and cardiometabolic diseases: A 2012 Korean Community Health Survey | nationwide/statewide study with no or insufficient area-specific adjustments |
| Lao et al. [12] | 2019 | Long-term exposure to ambient fine particulate matter (PM) and incident type 2 diabetes: a longitudinal cohort study | nationwide/statewide study with no or insufficient area-specific adjustments |
| Qiu et al. [13] | 2018 | Long-term exposure to fine particulate matter air pollution and type 2 diabetes mellitus in elderly: A cohort study in Hong Kong | spatial scale too crude (pollution surface): PM satellite data |
| Hansen et al. [14] | 2016 | Long-term exposure to fine particulate matter and incidence of diabetes in the Danish Nurse Cohort | nationwide/statewide study with no or insufficient area-specific adjustments |
| Liang et al. [15] | 2019 | Long-term exposure to ambient fine particulate matter and incidence of diabetes in China: A cohort study. | spatial scale too crude (pollution surface) |
| Jørgensen et al. [16] | 2019 | Long-Term Exposure to Road Traffic Noise and Incidence of Diabetes in the Danish Nurse Cohort. | nationwide/statewide study with no or insufficient area-specific adjustments |
| Liu et al. [17] | 2019 | Gut microbiota partially mediates the effects of fine particulate matter on type 2 diabetes: Evidence from a population-based epidemiological study. | nationwide/statewide study with no or insufficient area-specific adjustments |
| Kloog et al. [18] | 2012 | Acute and chronic effects of particles on hospital admissions in New-England | spatial scale too crude (pollution surface) |
| Sørensen et al. [19] | 2013 | Long-term exposure to road traffic noise and incident diabetes: a cohort study | other: no relevant exposure metric |
| Liu et al. [20] | 2016 | Associations between long-term exposure to ambient particulate air pollution and type 2 diabetes prevalence, blood glucose and glycosylated hemoglobin levels in China | spatial scale too crude (pollution surface) |
| Hart et al. [21] | 2015 | Effect Modification of Long-Term Air Pollution Exposures and the Risk of Incident Cardiovascular Disease in US Women | nationwide/statewide study with no or insufficient area-specific adjustments |
| Coogan et al. [22] | 2016 | PM2.5 and Diabetes and Hypertension Incidence in the Black Women's Health Study | nationwide/statewide study with no or insufficient area-specific adjustments |
| Hellack et al. [23] | 2017 | Land use regression modeling of oxidative potential of fine particles, NO2, PM2.5 mass and association to type two diabetes mellitus | review, methodological, HIA, or similar paper (no primary data) |
| Heidemann et al. [24] | 2014 | Residential traffic and incidence of Type 2 diabetes: the German Health Interview and Examination Surveys | other: self-reported exposure |
| Meo et al. [25] | 2015 | Effect of environmental air pollution on type 2 diabetes mellitus | review, methodological, HIA, or similar paper (no primary data) |
| Brook et al. [26] | 2008 | The relationship between diabetes mellitus and traffic-related air pollution | Very selective subgroup |
| Weaver et al. [27] | 2019 | Neighborhood sociodemographic effects on the associations between long-term PM exposure and cardiovascular outcomes and diabetes. | Very selective subgroup |
| Yang et al. [28] | 2018 | Ambient fine particulate pollution associated with diabetes mellitus among the elderly aged 50 years and older in China | no within-area or spatial contrast exploited |
| **Excluded studies from updated search with reasons** | | | |
| Thacher et al.[29] | 2021 | Long-Term Exposure toTransportation Noise and Risk for Type 2 Diabetes in a Nationwide Cohort Study from Denmark | no or insufficient area-specific adjustments |
| Jalali et al. [30] | 2021 | Long-term exposure to PM2.5 and cardiovascular disease incidence and mortality in an Eastern Mediterranean country: findings based on a 15-year cohort study | Other: traffic related measures did not end up in the final model |
| Meroni et al. [31] | 2021 | The relationship between air pollution and diabetes: A study on the municipalities of the Metropolitan City of Milan | Geographic study, spatial scale too crude (pollution surface) |
| Sørensen et al. [32] | 2022 | Air pollution, road traffic noise and lack of greenness and risk of type 2 diabetes: A multi-exposure prospective study covering Denmark | nationwide/statewide study with no or insufficient area-specific adjustments |
| Ye et al. [33] | 2021 | Association of long-term exposure to PM2.5 with hypertension and diabetes among the middle-aged and elderly people in Chinese mainland: a spatial study | spatial scale too crude (pollution surface) |
| Zhang et al. [34] | 2021 | Associations of long-term exposure to ambient nitrogen dioxide with indicators of diabetes and dyslipidemia in China: A nationwide analysis | nationwide/statewide study with no or insufficient area-specific adjustments |
| Paul et al. [35] | 2021 | The impact of air pollution on the incidence of diabetes and survival among prevalent diabetes cases | nationwide/statewide study with no or insufficient area-specific adjustments |
| Liu et al. [36] | 2019 | Gut microbiota partially mediates the effects of fine particulate matter on type 2 diabetes: Evidence from a population-based epidemiological study | nationwide/statewide study with lack of detail on the area adjustment |
| Li et al. [37] | 2019 | Association Between Long-term Exposure to PM2.5 and Incidence of Type 2 Diabetes in Taiwan: A National Retrospective Cohort Study | nationwide/statewide study with no or insufficient area-specific adjustments |
| Klompmaker et al. [38] | 2019 | Associations of Combined Exposures to Surrounding Green, Air Pollution, and Road Traffic Noise with Cardiometabolic Diseases | nationwide/statewide study with no or insufficient area-specific adjustments |
| Jorgensen et al. [16] | 2019 | Long-Term Exposure to Road Traffic Noise and Incidence of Diabetes in the Danish Nurse Cohort. | nationwide/statewide study with no or insufficient area-specific adjustments |
| Dimakakou et al. [39] | 2020 | Is Environmental and Occupational Particulate Air Pollution Exposure Related to Type-2 Diabetes and Dementia? A Cross-Sectional Analysis of the UK Biobank | nationwide/statewide study with no or insufficient area-specific adjustments |
| Li et al. [40] | 2021 | Obesity and the relation between joint exposure to ambient air pollutants and incident type 2 diabetes: A cohort study in UK Biobank | nationwide/statewide study with no or insufficient area-specific adjustments |

# Equation S1 For conversion of effect estimates to a standardized increment of exposure

For the re-scale, we assumed a log-linear shape of the CRF, as used in a recent air pollution health risk assessment by Khomenko et al. [41]), applying Equation:

| $\boldsymbol{RR}_{\boldsymbol{E}}\boldsymbol{=}\boldsymbol{e}^{\mathbf{ln}\left( \boldsymbol{RR}_{\boldsymbol{U}} \right)\boldsymbol{*}\frac{\boldsymbol{C}_{\boldsymbol{E}}}{\boldsymbol{U}}}$ | RR_U_ = Relative risk for a concentration D as in original literature.  U = Unit of concentration of the relative risk as in the original literature (e.g. 10 in μg/m^3^ PM_2.5_).  C_E_ = Desired increment of exposure, e.g. per 5 in μg/m^3^ PM_2.5_ |
| --- | --- |

We converted to common exposure units, that reflect a realistic range: per 10 µg/m3 for NO_2_, 20 µg/m^3^ for NO_x_, 1 µg/m^3^ for EC, 10 µg/m^3^ for PM_10_, and 5 µg/m^3^ for PM_2.5_.

# Figure S1 Assessing confidence in the quality of the body of evidence following OHAT [42]


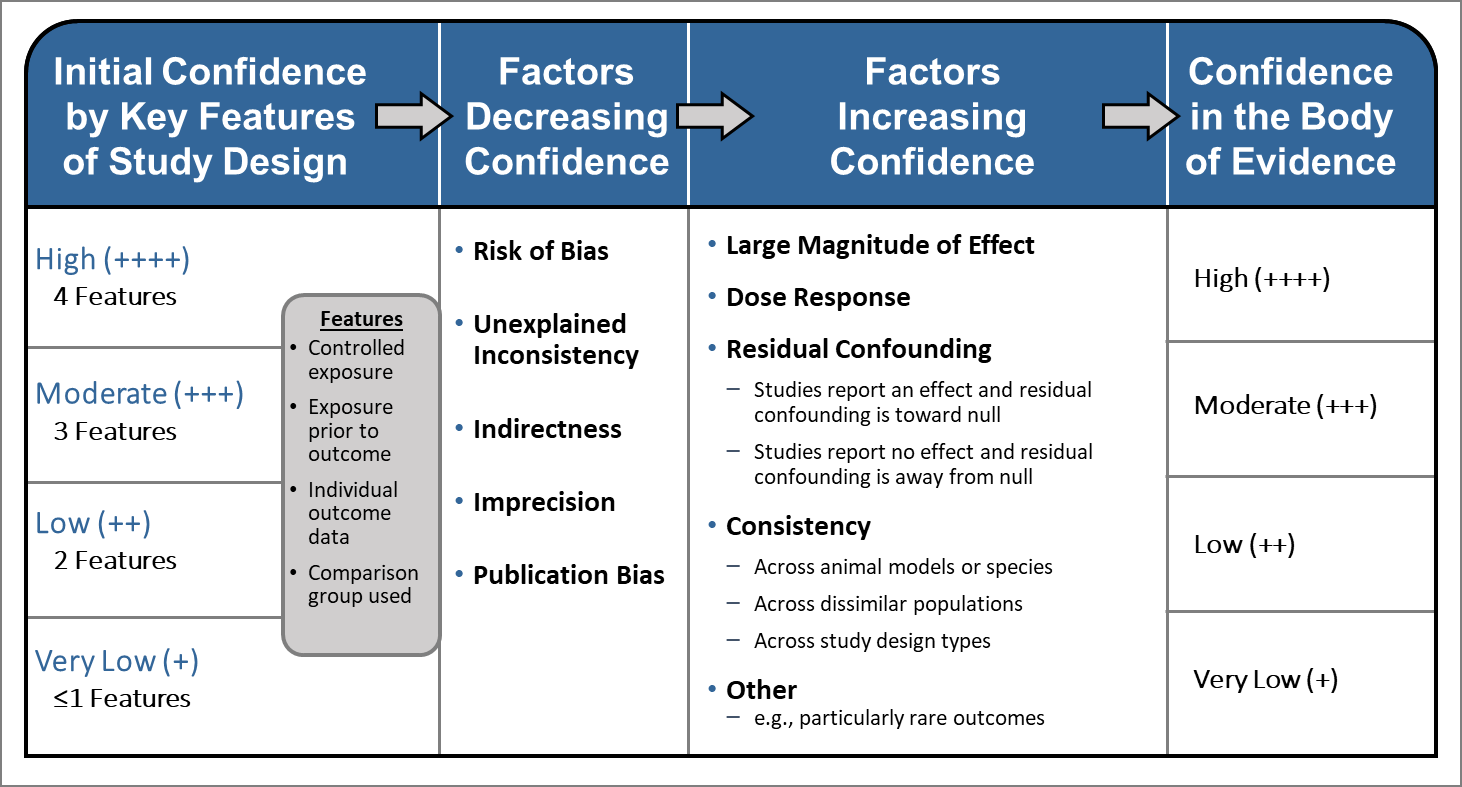


# Figure S2 Preferred Reporting Items For Systematic Reviews and Meta-Analysis (PRISMA) flow diagram for the search of the comprehensive review on the association of TRAP with various health outcomes with the focus on diabetes, search up to July 2019. (Global. 2022)


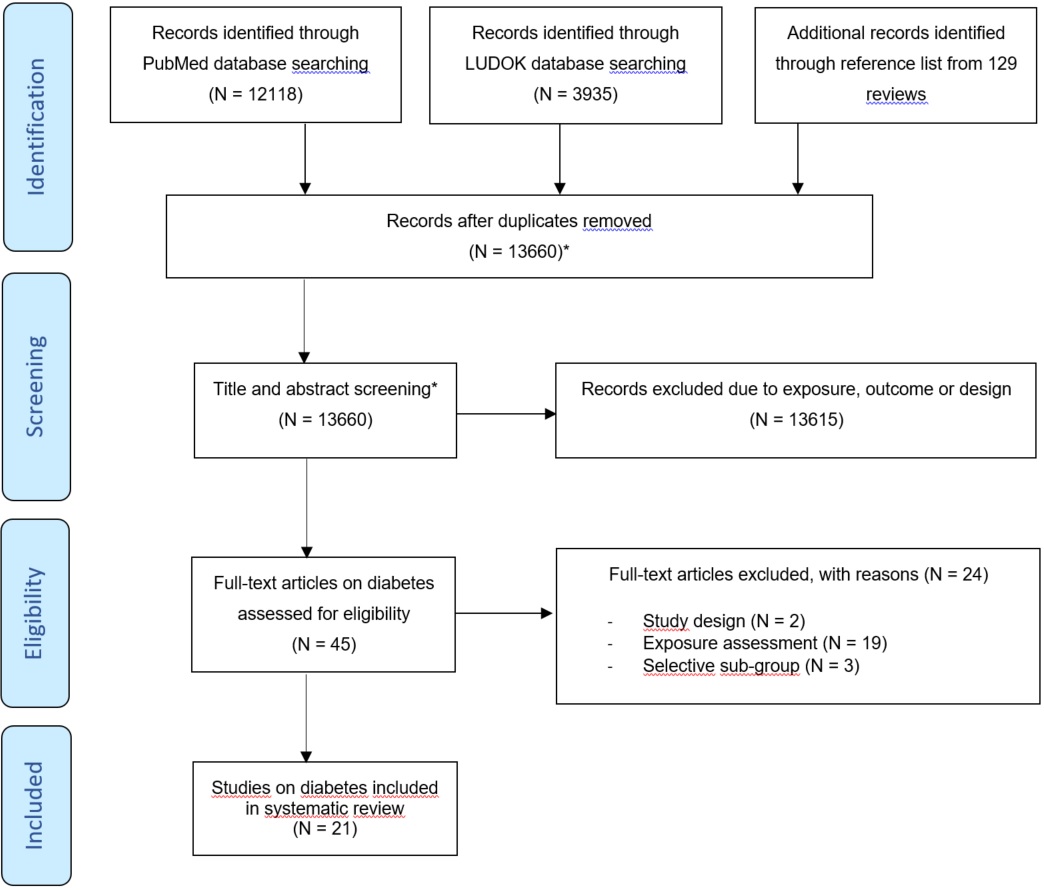


*Results of the comprehensive search including mortality, respiratory diseases, birth outcomes, and cardiometabolic health effects

# Figure S3 Forest-plot of the associations between distance measures and diabetes. (Global. 2022)

*SALIA estimates correspond to low and high education correspondingly

**Distance measures - Diabetes morbidity**

**Reference**

Kramer et al. 2010 [2]

Kramer et al. 2010

Puett et al. 2011 [1]

Puett et al. 2011

Puett et al. 2011

Andersen et al. 2012 [4]

Park et al. 2015 [4]

Weinmayr et al. 2015 [3]

Dijkema et al. 2011 [2]

Dijkema et al. 2011

Dijkema et al. 2011

Park et al. 2015

**Study Name**

SALIA

SALIA

Nurses' Health / Health Professionals Follow-Up

Nurses' Health / Health Professionals Follow-Up

Nurses' Health / Health Professionals Follow-Up

DDCH

MESA

HNR

Hoorn Diabetes Screening

Hoorn Diabetes Screening

Hoorn Diabetes Screening

MESA

**Measure**

Incidence

Incidence

Incidence

Incidence

Incidence

Incidence

Incidence

Incidence

Prevalence

Prevalence

Prevalence

Prevalence

**Categories**

<100 vs. >100 m

<100 vs. >100 m

0-49 vs. >200 m

50-99 vs. >200 m

100-199 vs. >200 m

<50 vs. >50 m

<100 vs. >100 m

<100 vs. 100-200 m

2-74 vs. 220-1610 m

74-140 vs. 220-1610 m

140-220 vs. 220-1610 m

<100 vs. >100 m

**RR**

2.54

0.92

1.11

0.96

0.96

1.07

0.96

1.37

0.88

1.17

1.12

1.10

**95% CI**

[1.31, 4.91]

[0.58, 1.47]

[1.01, 1.23]

[0.63, 1.48]

[0.87, 1.06]

[0.95, 1.21]

[0.80, 1.16]

[1.04, 1.81]

[0.70, 1.13]

[0.93, 1.48]

[0.88, 1.42]

[0.91, 1.34]

0

1

2

Relative Risk

# Figure S4 Forest-plot of the associations between traffic density measures and diabetes. (Gobal. 2022)

**Traffic Density measures - Diabetes morbidity**

**Reference**

Andersen et al. 2012 [4]

Dijkema et al. 2011 [2]

Dijkema et al. 2011 [2]

Dijkema et al. 2011 [2]

**Study Name**

DDCH

Hoorn Diabetes Screening

Hoorn Diabetes Screening

Hoorn Diabetes Screening

**Measure**

Incidence

Prevalence

Prevalence

Prevalence

**Increment/Categories**

per 1200 vehicles km/day

882-2007 vs. 63-516 thousand vehicles/day

680-882 vs. 63-516 thousand vehicles/day

516-680 vs. 63-516 thousand vehicles/day

**RR**

1.02

1.09

1.13

1.25

**95% CI**

[1.00, 1.04]

[0.85, 1.38]

[0.89, 1.44]

[0.99, 1.59]

0.5

1

1.5

Relative Risk

# Table S5 Risk of bias assessment for studies included in meta-analysis: diabetes. (Global. 2022)

| **Reference** | **Study Name** | **Confounding** | **Selection Bias** | **Exposure Assessment** | **Outcome Measurement** | **Missing Data** | **Selective Reporting** |
| --- | --- | --- | --- | --- | --- | --- | --- |
| Andersen, 2012 [4] | DDCH | Low | Low | Mod | Low | Low | Low |
| Bai, 2018 [43] | ONPHEC | High | Low | Mod | Low | Low | Low |
| Clark, 2017 [44] | British Columbia Diabetes Cohort | High | Low | Low | Low | Low | Low |
| Coogan, 2012 [45] | BWHS | Mod | Low | Low | Mod | Low | Low |
| Coogan, 2016 [46] | BWHS | Low | Low | Low | Mod | Mod | Low |
| Eze, 2014 [47] | SAPALDIA | Low | Mod | Low | Low | Low | Low |
| Eze, 2017 [48] | SAPALDIA | Low | High | Low | Low | Low | Low |
| Howell, 2019 [49] | CANHEART | High | Low | Low | Low | Low | Low |
| Kramer, 2010 [50] | SALIA | Mod | Low | Mod | Low | Mod | Low |
| Lazarevic, 2015 [51] | ALSWH | Low | Mod | Low | Mod | Low | Low |
| O'Donovan, 2017 [52] | CHAMPIONS | Low | Mod | Low | Low | Low | Low |
| Park, 2015 [53] | MESA | Low | Low | Mod | Low | Low | Low |
| Renzi, 2018 [54] | Rome Longitudinal | High | Low | Low | Low | Low | Low |
| Riant, 2018 [55] | ELISABET | Low | Low | Low | Low | Low | Low |
| Weinmayr, 2015 [3] | HNR | Low | Low | Low | Low | Low | Low |
| Yang, 2019 [56] | 33 CCHS | Low | Low | Low | Low | High | Low |

# Table S6 Results of the subgroup and sensitivity analysis for the diabetes prevalence. (Global. 2022)

Analysis were only conducted when three or more studies were available.

|  | | Prevalence of Diabetes | | | | | | | | |
| --- | --- | --- | --- | --- | --- | --- | --- | --- | --- | --- |
|  |  | NO_2_ | | | PM_10_ | | | PM_2.5_ | | |
| Stratification by |  | No. of studies | HR/OR (95%-CI) | Heterogeneity I2; T2; P-value | No. of studies | HR/OR (95%-CI) | Heterogeneity I2; T2; P-value | No. of studies | HR/OR (95%-CI) | Heterogeneity I2; T2; P-value |
| **None** |  | **7** | **1.09 [1.02; 1.17]** | **98%; 0.0043; p<0.01** | **4** | **1.19 [0.87: 1.63]** | **84%; 0.0433; p<0.01** | **3** | **1.08 [0.70; 1.67]** | **32%; 0.0213; p=0.23** |
| Region | North America | 1 | 1.08 [1.07; 1.09] | NA |  |  |  | 1 | NA |  |
|  | Western Europe | 4 | 1.08 [0.94; 1.25] | 64%; 0.0067; p=0.04 | 4 | 1.19 [0.87: 1.63] | 84%; 0.0433; p<0.01 | 2 | NA |  |
|  | Asia | 1 | 1.20 [1.09; 1.33] | NA |  |  |  |  |  |  |
|  | Australia/NZ | 1 | 1.06 [0.87; 1.29] | NA |  |  |  |  |  |  |
| Traffic Specificity | high | 6 | 1.07 [1.00; 1.15] | 98%; 0.0030, p<0.01 |  |  |  | 0 |  |  |
|  | moderate | 1 | 1.20 [1.09; 1.33] | NA | 4 | 1.19 [0.87: 1.63] | 84%; 0.0433; p<0.01 | 3 | 1.08 [0.70; 1.67] | 32%; 0.0213; p=0.23 |
| Selection bias | low | 4 | 1.08 [0.95; 1.23] | 99%; 0.055, p<0.01 | 2 | 0.99 [0.96;1.03] | 0%; 0.0; p=0.67 | 2 | NA |  |
|  | moderate/high | 3 | 1.14 [0.96; 1.36] | 0%, 0; p=0.50 | 2 | 1.43 [1.12; 1.83] | 0%; 0.0; p=0.82 | 1 |  |  |
| Smoking adjustment | yes | 5 | 1.17 [1.09; 1.25] | 0%; 0, p=0.74 | 3 | 1.43 [1.28; 1.59] | 0%; 0; 0.92 | 2 | NA |  |
|  | No | 2 | 1.04 [0.64; 1.70] |  | 1 | 0.99 [0.98; 1.00] | NA | 1 |  |  |
| Missing data RoB | low | 6 | 1.07 [1.00; 1.15] | 98%; 0.0030; p<0.01 | 4 | 1.19 [0.87: 1.63] | 84%; 0.0433; p<0.01 |  | NA |  |
|  | high | 1 | 1.20 [1.09; 1.33] | NA |  |  |  |  |  |  |
| Confounding | low/moderate | 5 | 1.17 [1.09; 1.25] | 0%; 0, p=0.74 | 3 | 1.43 [1.28; 1.59] | 0%; 0; 0.92 | 2 | NA |  |
|  | high | 2 | 1.04 [0.64; 1.70] |  | 1 | 0.99 [0.98; 1.00] | NA | 1 |  |  |
| RoB exposure assessment | low |  | NA |  |  | NA |  | 2 | NA |  |
|  | moderate |  |  |  |  |  |  | 1 |  |  |
| RoB outcome assessment | low | 6 | 1.10 [1.01; 1.19] | 98%; 0.0050; p<0.01 |  | NA |  | 2 | NA |  |
|  | moderate | 1 | 1.06 [0.87; 1.29] | NA |  |  |  | 1 |  |  |

The following increments were used: 10 µg/m3 for NO_2_, 20 µg/m^3^ for NO_x_, 1 µg/m^3^ for EC, 10 µg/m^3^ for PM_10_, and 5 µg/m^3^ for PM_2.5_. Effect estimates cannot be directly compared across the different traffic-related pollutants because the selected increments do not necessarily represent the same contrast in exposure.

# Table S7 Results of the subgroup and sensitivity analysis for the diabetes incidence. (Global. 2022)

Analysis were only conducted when three or more studies were available.

|  | | Incidence of Diabetes | | | | | | | | | | | |
| --- | --- | --- | --- | --- | --- | --- | --- | --- | --- | --- | --- | --- | --- |
|  |  | NO_2_ | | | NO_x_ | | | EC | | | PM_2.5_ | | |
| Stratification by |  | No. of studies | HR/OR (95%-CI) | Heterogeneity I2; T2; P-value | No. of studies | HR/OR (95%-CI) | Heterogeneity I2; T2; P-value | No. of studies | HR/OR (95%-CI) | Heterogeneity I2; T2; P-value | No. of studies | HR/OR (95%-CI) | Heterogeneity I2; T2; P-value |
| **None** |  | **7** | **1.04 [0.96; 1.17]** | **98%; 0.0051; p<0.01** | **4** | **1.02 [0.96; 1.10]** | **68%; 0.0003; p<0.03** | **3** | **1.16 [0.57; 2.36]** | **88%; 0.0612; p<0.01** | **4** | **1.05 [0.96; 1.15]** | **64%, 0.0030, p=0.04** |
| Region | North America | 3 | 1.01 [0.85; 1.19] | 96%; 0.0042; p<0.01 | 2 | NA |  | 1 | NA |  | 2 | NA |  |
|  | Western Europe | 4 | 1.07 [0.89; 1.29] | 81%; 0.0089; p<0.01 | 2 | NA |  | 2 | NA |  | 2 | NA |  |
|  | Asia | 0 |  |  |  |  |  |  |  |  |  |  |  |
|  | Australia/NZ | 0 |  |  |  |  |  |  |  |  |  |  |  |
| Traffic Specificity | high | 6 | 1.05 [0.97; 1.15] | 96%; 0.0038, p<0.01 | 4 | 1.02 [0.96; 1.10] | 68%; 0.0003; p<0.03 | 3 | 1.16 [0.57; 2.36] | 88%; 0.0612; p<0.01 | 0 |  |  |
|  | moderate | 1 | 0.94 [0.89; 1.00] | NA | 0 |  |  | 0 |  |  | 4 | 1.05 [0.96; 1.15] | 64%, 0.0030, p=0.04 |
| Selection bias | low | 6 | 1.04 [0.95; 1.15] | 96%; 0.0057; p<0.01 | 6 | 1.04 [0.95; 1.15] | 96%; 0.0057; p<0.01 | 3 | 1.16 [0.57; 2.36] | 88%; 0.0612; p<0.01 | 0 |  |  |
|  | moderate/high | 1 | 0.95 [0.77; 1.17] | NA | 1 | 0.95 [0.77; 1.17] | NA | 0 |  |  | 4 | 1.05 [0.96; 1.15] | 64%, 0.0030, p=0.04 |
| Smoking adjustment | yes | 4 | 1.05 [0.85; 1.31] | 85%; 0.0146; p<0.01 | 3 | 1.07 [0.82; 1.40] | 67%; 0.0069; p<0.05 | 1 | NA |  | 2 | 1.13 [0.84; 1.53] | 0%, 0, p=0.87 |
|  | No | 3 | 1.03 [0.92; 1.15] | 98%; 0.0019; p<0.01 | 1 | 1.01 [1.00; 1.02] | NA | 2 | NA |  | 2 | 1.04 [0.59; 1.85] | 87%, 0.0036, p<0.01 |
| Missing data RoB | low | 7 | 1.04 [0.96; 1.17] | 98%; 0.0051; p<0.01 | 4 | 1.02 [0.96; 1.10] | 68%; 0.0003; p<0.03 | 3 | 1.16 [0.57; 2.36] | 88%; 0.0612; p<0.01 | 0 |  |  |
|  | high | 0 |  |  | 0 |  |  | 0 |  |  | 4 | 1.05 [0.96; 1.15] | 64%, 0.0030, p=0.04 |
| Confounding | low/moderate | 4 | 1.05 [0.85; 1.31] | 85%; 0.0146; p<0.01 | 3 | 1.07 [0.82; 1.40] | 67%; 0.0069; p<0.05 | 1 | NA |  | 2 | 1.13 [0.84; 1.53] | 0%, 0, p=0.87 |
|  | high | 3 | 1.03 [0.92; 1.15] | 98%; 0.0019; p<0.01 | 1 | 1.01 [1.00; 1.02] | NA | 2 | NA |  | 2 | 1.04 [0.59; 1.85] | 87%, 0.0036, p<0.01 |
| RoB exposure assessment | low | 4 | 1.00 [0.99; 1.02] | 40%; 0.0; p=0.17 | 2 | 1.11 [0.27; 4.51] | 86%; 0.0214; p<0.01 | 2 | NA |  | 3 | 1.05 [0.90; 1.21] | 75%, 0.0033; p=0.02 |
|  | moderate | 3 | 1.11 [0.93; 1.34] | 62%; 0.0028; p=0.07 | 2 | 1.03 [0.92; 1.15] | 0%; 0.0; p=0.060 | 1 | NA |  | 1 | 1.11 [0.76; 1.62] | NA |
| RoB outcome assessment | low | 5 | 1.03 [0.98; 1.09] | 96%; 0.0016; p<0.01 | 3 | 1.01 [0.98; 1.05] | 4%; 0.0001; p=0.35 |  | NA |  |  | NA |  |
|  | moderate | 2 | 1.08 [0.17; 6.90] | 94%; 0.0398; p<0.01 | 1 | 1.26 [1.07; 1.48] | NA |  |  |  |  | NA |  |

The following increments were used: 10 µg/m3 for NO_2_, 20 µg/m^3^ for NO_x_, 1 µg/m^3^ for EC, 10 µg/m^3^ for PM_10_, and 5 µg/m^3^ for PM_2.5_. Effect estimates cannot be directly compared across the different traffic-related pollutants because the selected increments do not necessarily represent the same contrast in exposure.

# Table S8 Multi-pollutant analyses in diabetes studies considering noise. (Gobal. 2022)

| **Reference** | **Study Name** | **Pollutant** | **Incidence or prevalence** | **Effect measure** | **Increment** | **Single pollutant results** | **Noise adjusted** |
| --- | --- | --- | --- | --- | --- | --- | --- |
| Clark, 2017 [44] | British Columbia Diabetes Cohort | NO | Incidence | odds ratio (OR) | 13.13 μg/m^3^ | 1.04 (1.01, 1.05) | 1.01 (1.00, 1.04) |
| Clark, 2017 [44] | British Columbia Diabetes Cohort | PM_2.5 abs_ | Incidence | odds ratio (OR) | 0.9 1e-5/m | 1.03 (1.01, 1.04) | 1.01 (0.99, 1.03) |
| Clark, 2017 [44] | British Columbia Diabetes Cohort | PM_2.5_ | Incidence | odds ratio (OR) | 1.6 μg/m^3^ | 1.03 (1.01, 1.05) | 1.03 (1.02, 1.05) |
| Dzhambov, 2016 [57] | Plovdiv Diabetes Survey | PAH (BaP) | Prevalence | odds ratio (OR) | >6 vs. <6 ng/m3 | 1.76 (0.52, 5.98)^1^ | 1.76 (0.52, 5.98)^1^ |
| Dzhambov, 2016 [57] | Plovdiv Diabetes Survey | PM_2.5_ | Prevalence | odds ratio (OR) | >25 vs. <25 μg/m^3^ | 1.32 (0.28, 6.24)^1^ | 1.32 (0.28, 6.24)^1^ |
| Eze, 2014 [47] | SAPALDIA | NO_2_ | Prevalence | odds ratio (OR) | 10 μg/m^3^ | 1.21 (1.05, 1.39) | 1.19 (1.03, 1.38) |
| Eze, 2014 [47] | SAPALDIA | PM_10_ | Prevalence | odds ratio (OR) | 10 μg/m^3^ | 1.44 (1.21, 1.71) | 1.40 (1.17, 1.67) |
| Eze, 2017 [48] | SAPALDIA | NO_2_ | Incidence | relative risk (RR) | 15 μg/m^3^ | 0.92 (0.67, 1.26) | 0.86 (0.61, 1.22) |
| Renzi, 2018 [54] | Rome Longitudinal | NO_2_ | Incidence | hazard ratio (HR) | 10 μg/m^3^ | 1.00 (1.00, 1.01) | 1.00 (0.99, 1.01) |
| Renzi, 2018 [54] | Rome Longitudinal | NO_2_ | Prevalence | odds ratio (OR) | 10 μg/m^3^ | 1.00 (1.00, 1.01) | 1.01 (1.00, 1.02) |
| Renzi, 2018 [54] | Rome Longitudinal | NO_x_ | Incidence | hazard ratio (HR) | 20 μg/m^3^ | 1.01 (1.00, 1.01) | 1.01 (1.00, 1.02) |
| Renzi, 2018 [54] | Rome Longitudinal | NO_x_ | Prevalence | odds ratio (OR) | 20 μg/m^3^ | 1.01 (1.00, 1.01) | 1.02 (1.01, 1.02) |
| Renzi, 2018 [54] | Rome Longitudinal | PM_10_ | Incidence | hazard ratio (HR) | 10 μg/m^3^ | 1.00 (0.99, 1.02) | 1.00 (0.98, 1.02) |
| Renzi, 2018 [54] | Rome Longitudinal | PM_10_ | Prevalence | odds ratio (OR) | 10 μg/m^3^ | 0.99 (0.98, 1.00) | 1.00 (0.99, 1.02) |
| Renzi, 2018 [54] | Rome Longitudinal | PM_2.5 abs_ | Incidence | hazard ratio (HR) | 1 1e-5/m | 1.00 (0.98, 1.02) | 1.00 (0.98, 1.02) |
| Renzi, 2018 [54] | Rome Longitudinal | PM_2.5 abs_ | Prevalence | odds ratio (OR) | 1 1e-5/m | 0.98 (0.96, 0.99) | 0.98 (0.97, 1.00) |
| Renzi, 2018 [54] | Rome Longitudinal | PM_2.5_ | Incidence | hazard ratio (HR) | 5 μg/m^3^ | 1.00 (0.98, 1.02) | 1.00 (0.97, 1.02) |
| Renzi, 2018 [54] | Rome Longitudinal | PM_2.5_ | Prevalence | odds ratio (OR) | 5 μg/m^3^ | 0.98 (0.96, 1.00) | 0.92 (0.97, 1.01) |
| Renzi, 2018 [54] | Rome Longitudinal | PMcoarse mass | Incidence | hazard ratio (HR) | 10 μg/m^3^ | 0.99 (0.97, 1.02) | 0.98 (0.95, 1.01) |
| Renzi, 2018 [54] | Rome Longitudinal | PMcoarse mass | Prevalence | odds ratio (OR) | 10 μg/m^3^ | 0.96 (0.94, 0.98) | 0.97 (0.95, 0.99) |

^1^The single pollutant results also corrected for noise; hence the two columns are similar.

# Figure S5 Comparison of meta-analytic results of associations between traffic-related air pollutants and diabetes prevalence and incidence from original analyses including studies up to July 2019 (squares) and the updated analysis (triangles) including studies up to May 2022. (Global. 2023)


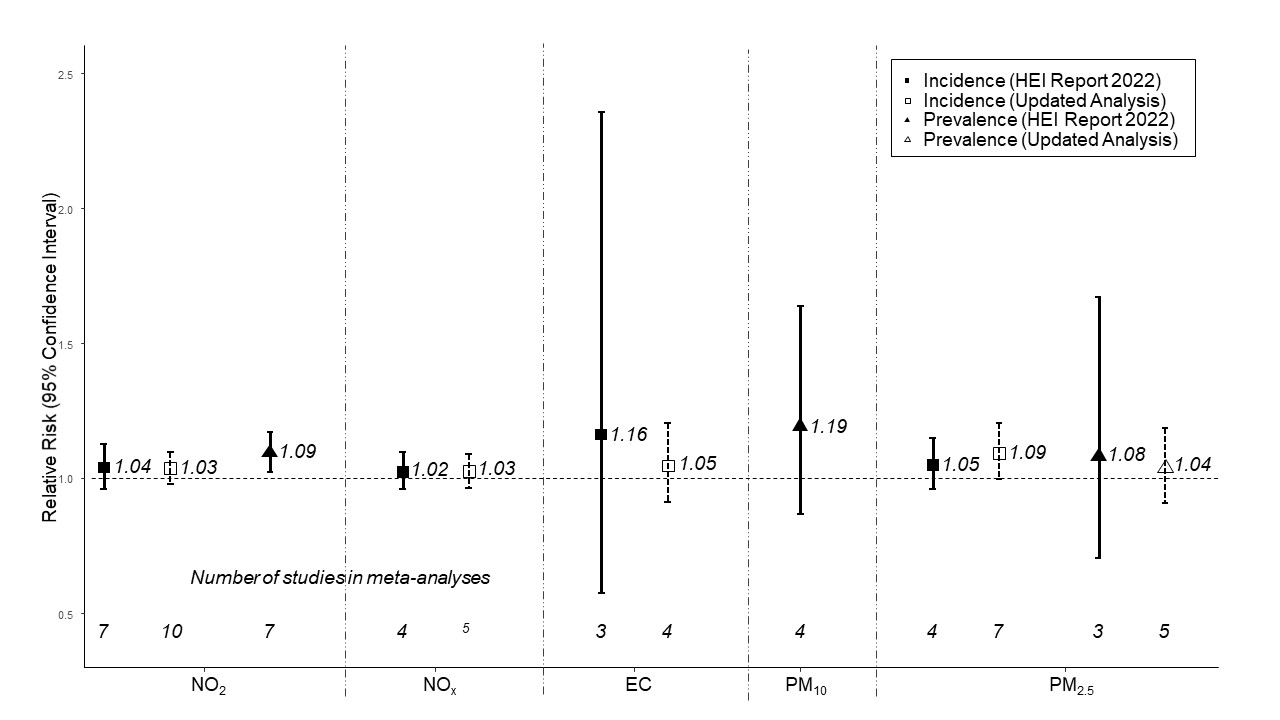


The following increments were used: 10 µg/m^3^ for NO_2_, 20 µg/m^3^ for NO_x_, 1 µg/m^3^ for EC, 10 µg/m^3^ for PM_10_, and 5 µg/m^3^ for PM_2.5_. Effect estimates cannot be directly compared across the different traffic-related pollutants because the selected increments do not necessarily represent the same contrast in exposure. No new studies were added from the update for the prevalence analysis with NO_2_ and PM_10_.

#
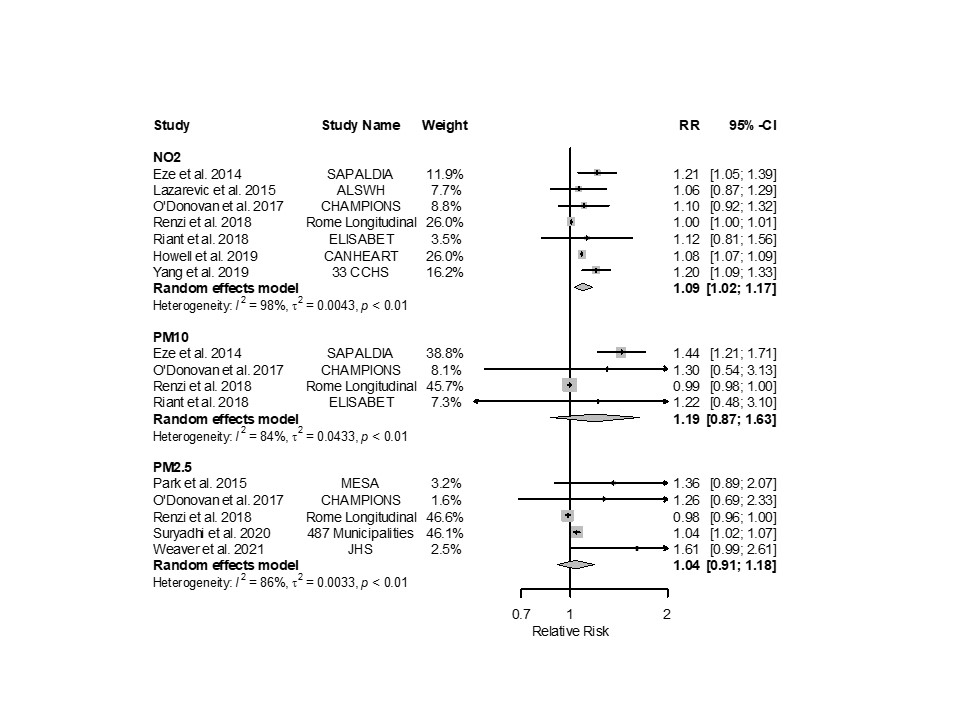
Figure S6 Forest plots of adjusted RRs (95% CIs) for diabetes prevalence with NO_2_, PM_10_ and PM_2.5_ from the updated analysis including studies up to May 2022. (Global. 2023)

The size of the grey squares represents the weight of the study in the meta-analysis. The following increments were used: 10 µg/m^3^ for NO_2_, 20 µg/m^3^ for NO_x_, 1 µg/m^3^ for EC, 10 µg/m^3^ for PM_10_, and 5 µg/m^3^ for PM_2.5_. Effect estimates cannot be directly compared across the different traffic-related pollutants because the selected increments do not necessarily represent the same contrast in exposure.

New study references Lucht [58], Yu [59], Sorensen[60], Weaver [61]

# Figure S7 Forest plots of adjusted RRs (95% CIs) for diabetes prevalence with NO_2_, NO_x_, EC, and PM_2.5_ from the updated analysis including studies up to May 2022. (Global. 2023)


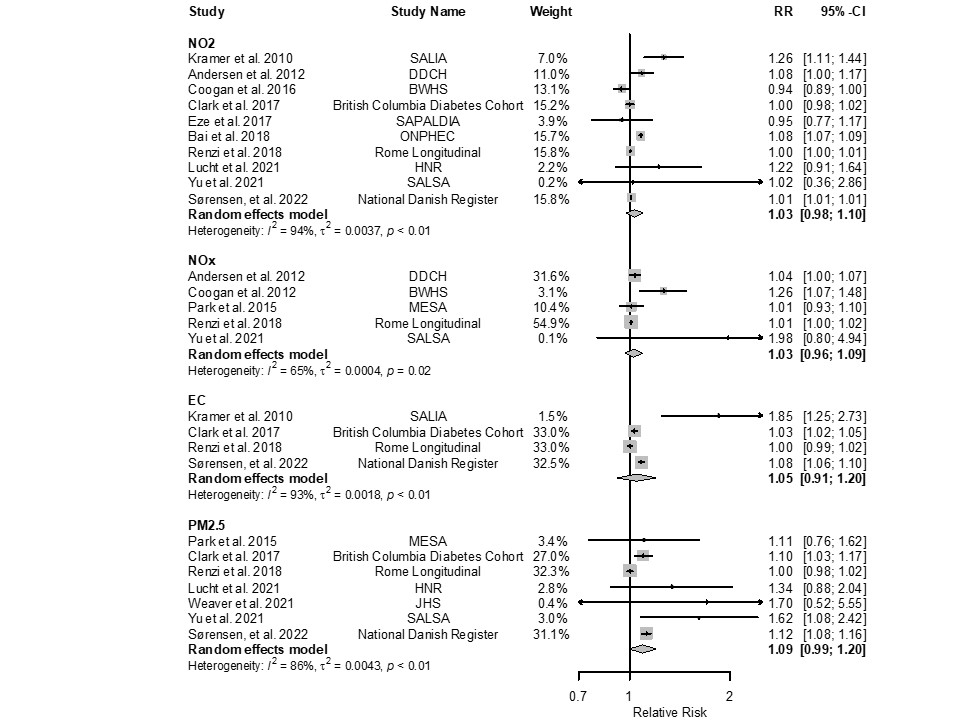


Note: only PM_2.5_ was updated

The size of the grey squares represents the weight of the study in the meta-analysis. The following increments were used: 10 µg/m^3^ for NO_2_, 20 µg/m^3^ for NO_x_, 1 µg/m^3^ for EC, 10 µg/m^3^ for PM_10_, and 5 µg/m^3^ for PM_2.5_. Effect estimates cannot be directly compared across the different traffic-related pollutants because the selected increments do not necessarily represent the same contrast in exposure.

New study reference Suryadi [62], Weaver [61]

# Table S9 Comparison of effect estimates with previously published reviews on diabetes prevalence and incidence with ambient air pollution.

| **Prevalence** | | | | | | |
| --- | --- | --- | --- | --- | --- | --- |
| Authors | PM_2.5_ per 10 μg/m^3^ | Number of studies | PM_10_ per 10 μg/m^3^ | Number of studies | NO_2_ per 10 μg/m^3^ | Number of studies |
| HEI 2022 [63] | 1.16 (0.49-2.79)* original: 1.08 (0.70-1.67) | 3 | 1.19 (0.87-1.63) | 4 | **1.09 (1.02-1.17)** | 7 |
| Liu 2019 [64] | **1.09 (1.05-1.13)** | 11 | **1.12 (1.06-1.13)** | 7 | **1.05 (1.03-1.08)** | 12 |
| Yang 2020 [65] | **1.08 (1.04-1.12)** | 11 | **1.10 (1.03-1.17)** | 6 | **1.07 (1.04-1.11)** | 11 |
| **Incidence** | | | | | | |
|  | PM_2.5_ per 10 μg /m^3^ | Number of studies | PM_10_ per 10 μg /m^3^ | Number of studies | NO_2_ per 10 μg/m^3^ | Number of studies |
| HEI 2022 [63] | 1.10 (0.92-1.32)* original 1.05 (0.96-1.15) | 4 | - |  | 1.04 (0.96-1.13) | 7 |
| Liu 2019 [64] | **1.10 (1.04-1.16)** | 12 | 1.05 (0.98-1.13) | 4 | 1.02 (0.99-1.05) | 9 |
| Yang 2020 [65] | **1.10 (1.04-1.17)** | 11 | **1.11 (1.00-1.22)** | **6** | 1.01 (0.99-1.02) | 7 |

* For the re-scale, we assumed a log-linear shape of the CRF, as in Khomenko et al. [41], applying Equation:

| $\boldsymbol{RR}_{\boldsymbol{E}}\boldsymbol{=}\boldsymbol{e}^{\mathbf{ln}\left( \boldsymbol{RR}_{\boldsymbol{U}} \right)\boldsymbol{*}\frac{\boldsymbol{C}_{\boldsymbol{E}}}{\boldsymbol{U}}}$ | RR_U_ = Relative risk for a concentration D as in literature.  U = Unit of concentration of the relative risk as in the literature (e.g. 10 in μg/m^3^ PM).  C_E_ = Desired increment of exposure |
| --- | --- |

# References

[1] Puett RC, Hart JE, Schwartz J, Hu FB, Liese AD, Laden F (2011) Are particulate matter exposures associated with risk of type 2 diabetes? Environ Health Perspect 119(3): 384-389. 10.1289/ehp.1002344

[2] Dijkema MB, Mallant SF, Gehring U, et al. (2011) Long-term exposure to traffic-related air pollution and type 2 diabetes prevalence in a cross-sectional screening-study in the Netherlands. Environ Health 10: 76. 10.1186/1476-069X-10-76

[3] Weinmayr G, Hennig F, Fuks K, et al. (2015) Long-term exposure to fine particulate matter and incidence of type 2 diabetes mellitus in a cohort study: effects of total and traffic-specific air pollution. Environ Health 14: 53. 10.1186/s12940-015-0031-x

[4] Andersen ZJ, Raaschou-Nielsen O, Ketzel M, et al. (2012) Diabetes incidence and long-term exposure to air pollution: a cohort study. Diabetes Care 35(1): 92-98. 10.2337/dc11-1155

[5] Requia WJ, Adams MD, Koutrakis P (2017) Association of PM2.5 with diabetes, asthma, and high blood pressure incidence in Canada: A spatiotemporal analysis of the impacts of the energy generation and fuel sales. Science of the Total Environment 584: 1077-1083. 10.1016/j.scitotenv.2017.01.166

[6] Strak M, Janssen N, Beelen R, et al. (2017) Long-term exposure to particulate matter, NO2 and the oxidative potential of particulates and diabetes prevalence in a large national health survey. Environment International 108: 228-236. 10.1016/j.envint.2017.08.017

[7] Orioli R, Cremona G, Ciancarella L, Solimini AG (2018) Association between PM10, PM2.5, NO2, O-3 and self-reported diabetes in Italy: A cross-sectional, ecological study. Plos One 13(1). 10.1371/journal.pone.0191112

[8] Hazlehurst MF, Nurius PS, Hajat A (2018) Individual and Neighborhood Stressors, Air Pollution and Cardiovascular Disease. Int J Env Res Pub He 15(3). 10.3390/ijerph15030472

[9] Bowe B, Xie Y, Li TT, Yan Y, Xian H, Al-Aly Z (2018) The 2016 global and national burden of diabetes mellitus attributable to PM2.5 air pollution. Lancet Planetary Health 2(7): E301-E312. Doi 10.1016/S2542-5196(18)30140-2

[10] Gandini M, Scarinzi C, Bande S, et al. (2018) Long term effect of air pollution on incident hospital admissions: Results from the Italian Longitudinal Study within LIFE MED HISS project. Environment International 121: 1087-1097. 10.1016/j.envint.2018.10.020

[11] Shin J, Choi J, Kim KJ (2019) Association between long-term exposure of ambient air pollutants and cardiometabolic diseases: A 2012 Korean Community Health Survey. Nutr Metab Cardiovas 29(2): 144-151. 10.1016/j.numecd.2018.09.008

[12] Lao XQ, Guo C, Chang LY, et al. (2019) Long-term exposure to ambient fine particulate matter (PM2.5) and incident type 2 diabetes: a longitudinal cohort study. Diabetologia 62(5): 759-769. 10.1007/s00125-019-4825-1

[13] Qiu H, Schooling CM, Sun SZ, et al. (2018) Long-term exposure to fine particulate matter air pollution and type 2 diabetes mellitus in elderly: A cohort study in Hong Kong. Environment International 113: 350-356. 10.1016/j.envint.2018.01.008

[14] Hansen AB, Ravnskjaer L, Loft S, et al. (2016) Long-term exposure to fine particulate matter and incidence of diabetes in the Danish Nurse Cohort. Environment International 91: 243-250. 10.1016/j.envint.2016.02.036

[15] Liang FC, Yang XL, Liu FC, et al. (2019) Long-term exposure to ambient fine particulate matter and incidence of diabetes in China: A cohort study. Environment International 126: 568-575. 10.1016/j.envint.2019.02.069

[16] Jorgensen JT, Brauner EV, Backalarz C, et al. (2019) Long-Term Exposure to Road Traffic Noise and Incidence of Diabetes in the Danish Nurse Cohort. Environ Health Persp 127(5). 10.1289/Ehp4389

[17] Liu T, Chen XJ, Xu YJ, et al. (2019) Gut microbiota partially mediates the effects of fine particulate matter on type 2 diabetes: Evidence from a population-based epidemiological study. Environment International 130. 10.1016/j.envint.2019.05.076

[18] Kloog I, Coull BA, Zanobetti A, Koutrakis P, Schwartz JD (2012) Acute and Chronic Effects of Particles on Hospital Admissions in New-England. Plos One 7(4). 10.1371/journal.pone.0034664

[19] Sorensen M, Andersen ZJ, Nordsborg RB, et al. (2013) Long-Term Exposure to Road Traffic Noise and Incident Diabetes: A Cohort Study. Environ Health Persp 121(2): 217-222. 10.1289/ehp.1205503

[20] Liu C, Yang CY, Zhao YH, et al. (2016) Associations between long-term exposure to ambient particulate air pollution and type 2 diabetes prevalence, blood glucose and glycosylated hemoglobin levels in China. Environment International 92-93: 416-421. 10.1016/j.envint.2016.03.028

[21] Hart JE, Puett RC, Rexrode KM, Albert CM, Laden F (2015) Effect Modification of Long-Term Air Pollution Exposures and the Risk of Incident Cardiovascular Disease in US Women. J Am Heart Assoc 4(12). 10.1161/JAHA.115.002301

[22] Coogan PF, White LF, Yu J, et al. (2016) PM2.5 and Diabetes and Hypertension Incidence in the Black Women's Health Study. Epidemiology 27(2): 202-210. 10.1097/Ede.0000000000000418

[23] Hellack B, Sugiri D, Schins RPF, et al. (2017) Land use regression modeling of oxidative potential of fine particles, NO2, PM2.5 mass and association to type two diabetes mellitus. Atmospheric Environment 171: 181-190. 10.1016/j.atmosenv.2017.10.017

[24] Heidemann C, Niemann H, Paprott R, Du Y, Rathmann W, Scheidt-Nave C (2014) Residential traffic and incidence of Type 2 diabetes: the German Health Interview and Examination Surveys. Diabetic Med 31(10): 1269-1276. 10.1111/dme.12480

[25] Meo SA, Memon AN, Sheikh SA, et al. (2015) Effect of environmental air pollution on type 2 diabetes mellitus. Eur Rev Med Pharmacol Sci 19(1): 123-128

[26] Brook RD, Jerreft M, Brook JR, Bard RL, Finkelstein MM (2008) The relationship between diabetes mellitus and traffic-related air pollution. J Occup Environ Med 50(1): 32-38. 10.1097/JOM.0b013e31815dba70

[27] Weaver AM, McGuinn L, Neas L, et al. (2019) Neighborhood sociodemographic effects on the associations between long-term PM(2.5) exposure and cardiovascular outcomes and diabetes. Environ Epidemiol 3(1). 10.1097/ee9.0000000000000038

[28] Yang Y, Guo YF, Qian ZM, et al. (2018) Ambient fine particulate pollution associated with diabetes mellitus among the elderly aged 50 years and older in China. Environmental Pollution 243: 815-823. 10.1016/j.envpol.2018.09.056

[29] Thacher JD, Poulsen AH, Hvidtfeldt UA, et al. (2021) Long-Term Exposure to Transportation Noise and Risk for Type 2 Diabetes in a Nationwide Cohort Study from Denmark. Environ Health Perspect 129(12): 127003. 10.1289/ehp9146

[30] Jalali S, Karbakhsh M, Momeni M, et al. (2021) Long-term exposure to PM(2.5) and cardiovascular disease incidence and mortality in an Eastern Mediterranean country: findings based on a 15-year cohort study. Environ Health 20(1): 112. 10.1186/s12940-021-00797-w

[31] Meroni G, Valerio A, Vezzoli M, Croci E, Carruba MO (2021) The relationship between air pollution and diabetes: A study on the municipalities of the Metropolitan City of Milan. Diabetes Res Clin Pract 174: 108748. 10.1016/j.diabres.2021.108748

[32] Sørensen M, Poulsen AH, Hvidtfeldt UA, et al. (2022) Air pollution, road traffic noise and lack of greenness and risk of type 2 diabetes: A multi-exposure prospective study covering Denmark. Environ Int 170: 107570. 10.1016/j.envint.2022.107570

[33] Ye Z, Li X, Han Y, Wu Y, Fang Y (2022) Association of long-term exposure to PM(2.5) with hypertension and diabetes among the middle-aged and elderly people in Chinese mainland: a spatial study. BMC Public Health 22(1): 569. 10.1186/s12889-022-12984-6

[34] Zhang Q, Liu C, Wang Y, et al. (2021) Associations of long-term exposure to ambient nitrogen dioxide with indicators of diabetes and dyslipidemia in China: A nationwide analysis. Chemosphere 269: 128724. <https://doi.org/10.1016/j.chemosphere.2020.128724>

[35] Paul LA, Burnett RT, Kwong JC, et al. (2020) The impact of air pollution on the incidence of diabetes and survival among prevalent diabetes cases. Environment International 134: 105333. <https://doi.org/10.1016/j.envint.2019.105333>

[36] Liu T, Chen X, Xu Y, et al. (2019) Gut microbiota partially mediates the effects of fine particulate matter on type 2 diabetes: Evidence from a population-based epidemiological study. Environ Int 130: 104882. 10.1016/j.envint.2019.05.076

[37] Li CY, Wu CD, Pan WC, Chen YC, Su HJ (2019) Association Between Long-term Exposure to PM2.5 and Incidence of Type 2 Diabetes in Taiwan: A National Retrospective Cohort Study. Epidemiology 30 Suppl 1: S67-s75. 10.1097/ede.0000000000001035

[38] Klompmaker JO, Janssen NAH, Bloemsma LD, et al. (2019) Associations of Combined Exposures to Surrounding Green, Air Pollution, and Road Traffic Noise with Cardiometabolic Diseases. Environ Health Perspect 127(8): 87003. 10.1289/ehp3857

[39] Dimakakou E, Johnston HJ, Streftaris G, Cherrie JW (2020) Is Environmental and Occupational Particulate Air Pollution Exposure Related to Type-2 Diabetes and Dementia? A Cross-Sectional Analysis of the UK Biobank. Int J Environ Res Public Health 17(24). 10.3390/ijerph17249581

[40] Li X, Wang M, Song Y, et al. (2021) Obesity and the relation between joint exposure to ambient air pollutants and incident type 2 diabetes: A cohort study in UK Biobank. PLoS Med 18(8): e1003767. 10.1371/journal.pmed.1003767

[41] Khomenko S, Cirach M, Pereira-Barboza E, et al. (2021) Premature mortality due to air pollution in European cities: a health impact assessment. The Lancet Planetary Health 5(3): e121-e134. <https://doi.org/10.1016/S2542-5196(20)30272-2>

[42] Office of Health Assessment and Translation O (2019) Handbook for Conducting a Literature-Based Health Assessment Using OHAT Approach for Systematic Review and Evidence Integration. U.S. Department of Health and Human Services

[43] Bai L, Chen H, Hatzopoulou M, et al. (2018) Exposure to Ambient Ultrafine Particles and Nitrogen Dioxide and Incident Hypertension and Diabetes. Epidemiology 29(3): 323-332. 10.1097/EDE.0000000000000798

[44] Clark C, Sbihi H, Tamburic L, Brauer M, Frank LD, Davies HW (2017) Association of Long-Term Exposure to Transportation Noise and Traffic-Related Air Pollution with the Incidence of Diabetes: A Prospective Cohort Study. Environ Health Perspect 125(8): 087025. 10.1289/EHP1279

[45] Coogan PF, White LF, Jerrett M, et al. (2012) Air pollution and incidence of hypertension and diabetes mellitus in black women living in Los Angeles. Circulation 125(6): 767-772. 10.1161/circulationaha.111.052753

[46] Coogan PF, White LF, Yu J, et al. (2016) Long term exposure to NO2 and diabetes incidence in the Black Women's Health Study. Environ Res 148: 360-366. 10.1016/j.envres.2016.04.021

[47] Eze IC, Schaffner E, Fischer E, et al. (2014) Long-term air pollution exposure and diabetes in a population-based Swiss cohort. Environ Int 70: 95-105. 10.1016/j.envint.2014.05.014

[48] Eze IC, Foraster M, Schaffner E, et al. (2017) Long-term exposure to transportation noise and air pollution in relation to incident diabetes in the SAPALDIA study. Int J Epidemiol 46(4): 1115-1125. 10.1093/ije/dyx020

[49] Howell NA, Tu JV, Moineddin R, et al. (2019) Interaction between neighborhood walkability and traffic-related air pollution on hypertension and diabetes: The CANHEART cohort. Environ Int 132: 104799. 10.1016/j.envint.2019.04.070

[50] Kramer U, Herder C, Sugiri D, et al. (2010) Traffic-related air pollution and incident type 2 diabetes: results from the SALIA cohort study. Environ Health Perspect 118(9): 1273-1279. 10.1289/ehp.0901689

[51] Lazarevic N, Dobson AJ, Barnett AG, Knibbs LD (2015) Long-term ambient air pollution exposure and self-reported morbidity in the Australian Longitudinal Study on Women's Health: a cross-sectional study. BMJ Open 5(10): e008714. 10.1136/bmjopen-2015-008714

[52] O'Donovan G, Chudasama Y, Grocock S, et al. (2017) The association between air pollution and type 2 diabetes in a large cross-sectional study in Leicester: The CHAMPIONS Study. Environ Int 104: 41-47. 10.1016/j.envint.2017.03.027

[53] Park SK, Adar SD, O'Neill MS, et al. (2015) Long-term exposure to air pollution and type 2 diabetes mellitus in a multiethnic cohort. Am J Epidemiol 181(5): 327-336. 10.1093/aje/kwu280

[54] Renzi M, Cerza F, Gariazzo C, et al. (2018) Air pollution and occurrence of type 2 diabetes in a large cohort study. Environ Int 112: 68-76. 10.1016/j.envint.2017.12.007

[55] Riant M, Meirhaeghe A, Giovannelli J, et al. (2018) Associations between long-term exposure to air pollution, glycosylated hemoglobin, fasting blood glucose and diabetes mellitus in northern France. Environ Int 120: 121-129. 10.1016/j.envint.2018.07.034

[56] Yang BY, Guo Y, Markevych I, et al. (2019) Association of Long-term Exposure to Ambient Air Pollutants With Risk Factors for Cardiovascular Disease in China. JAMA Netw Open 2(3): e190318. 10.1001/jamanetworkopen.2019.0318

[57] Dzhambov AM, Dimitrova DD (2016) Exposures to road traffic, noise, and air pollution as risk factors for type 2 diabetes: A feasibility study in Bulgaria. Noise Health 18(82): 133-142. 10.4103/1463-1741.181996

[58] Lucht S, Hennig F, Moebus S, et al. (2020) All-source and source-specific air pollution and 10-year diabetes Incidence: Total effect and mediation analyses in the Heinz Nixdorf recall study. Environ Int 136: 105493. 10.1016/j.envint.2020.105493

[59] Yu Y, Jerrett M, Paul KC, et al. (2021) Ozone Exposure, Outdoor Physical Activity, and Incident Type 2 Diabetes in the SALSA Cohort of Older Mexican Americans. Environ Health Perspect 129(9): 97004. 10.1289/ehp8620

[60] Sørensen M, Poulsen AH, Hvidtfeldt UA, et al. (2022) Exposure to source-specific air pollution and risk for type 2 diabetes: a nationwide study covering Denmark. International Journal of Epidemiology 51(4): 1219-1229. 10.1093/ije/dyac040

[61] Weaver AM, Bidulescu A, Wellenius GA, et al. (2021) Associations between air pollution indicators and prevalent and incident diabetes in an African American cohort, the Jackson Heart Study. Environ Epidemiol 5(3): e140. 10.1097/ee9.0000000000000140

[62] Suryadhi MAH, Suryadhi PAR, Abudureyimu K, et al. (2020) Exposure to particulate matter (PM(2.5)) and prevalence of diabetes mellitus in Indonesia. Environ Int 140: 105603. 10.1016/j.envint.2020.105603

[63] HEI (Health Effects Institute) (2022) Systematic Review and Meta-Analysis of Selected Health Effects of Long-Term Exposure to Traffic-Related Air Pollution. In. Health Effects Institute, Boston, MA, USA

[64] Liu F, Chen G, Huo W, et al. (2019) Associations between long-term exposure to ambient air pollution and risk of type 2 diabetes mellitus: A systematic review and meta-analysis. Environ Pollut 252(Pt B): 1235-1245. 10.1016/j.envpol.2019.06.033

[65] Yang BY, Fan S, Thiering E, et al. (2020) Ambient air pollution and diabetes: A systematic review and meta-analysis. Environ Res 180: 108817. 10.1016/j.envres.2019.108817
